# Supplementary material for: Bacterial outer membrane vesicles suppress tumor by interferon-γ-mediated antitumor response
Source: Nat Commun. 2017 Sep 20;8:626. doi: 10.1038/s41467-017-00729-8 (PMC5606984; doi:10.1038/s41467-017-00729-8)
Supplement: Supplementary file 1 — Supplementary Information [file 41467_2017_729_MOESM1_ESM.pdf]

Description of Supplementary Files

File Name: Supplementary Information
Description: Supplementary Figures, Supplementary Tables, Supplementary Methods and Supplementary References

File Name: Peer Review File

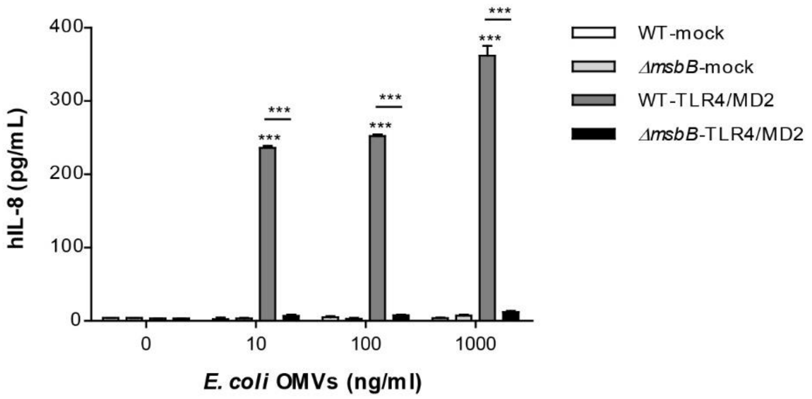

Supplementary Figure 1. *E. coli msbB* mutant (Δ*msbB*) OMVs have impaired lipid A function. Production of human IL-8 cytokines from mock- and TLR4/MD2-transfected human embryonic kidney HEK293 cells after *E. coli* WT and Δ*msbB* OMV-treatments (n = 4, two independent experiments). Data are presented as the mean ± s.d. from a representative experiments. *** indicates $P < 0.001$ analysed unpaired Student's t-test.

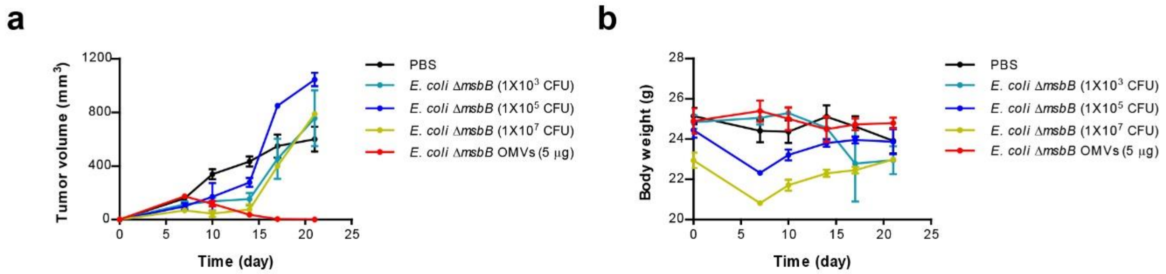

Supplementary Figure 2. Tumor regression effect of *E. coli* Δ*msbB* mutant bacteria. a,b, Tumor volume (a) and body weight (b) of mice bearing CT26 tumor measured after treatments of *E. coli* Δ*msbB* mutant bacteria and *E. coli* Δ*msbB* OMVs (*n* = 7 mice per group). Data are presented as the mean ± s.d. from a representative experiments.

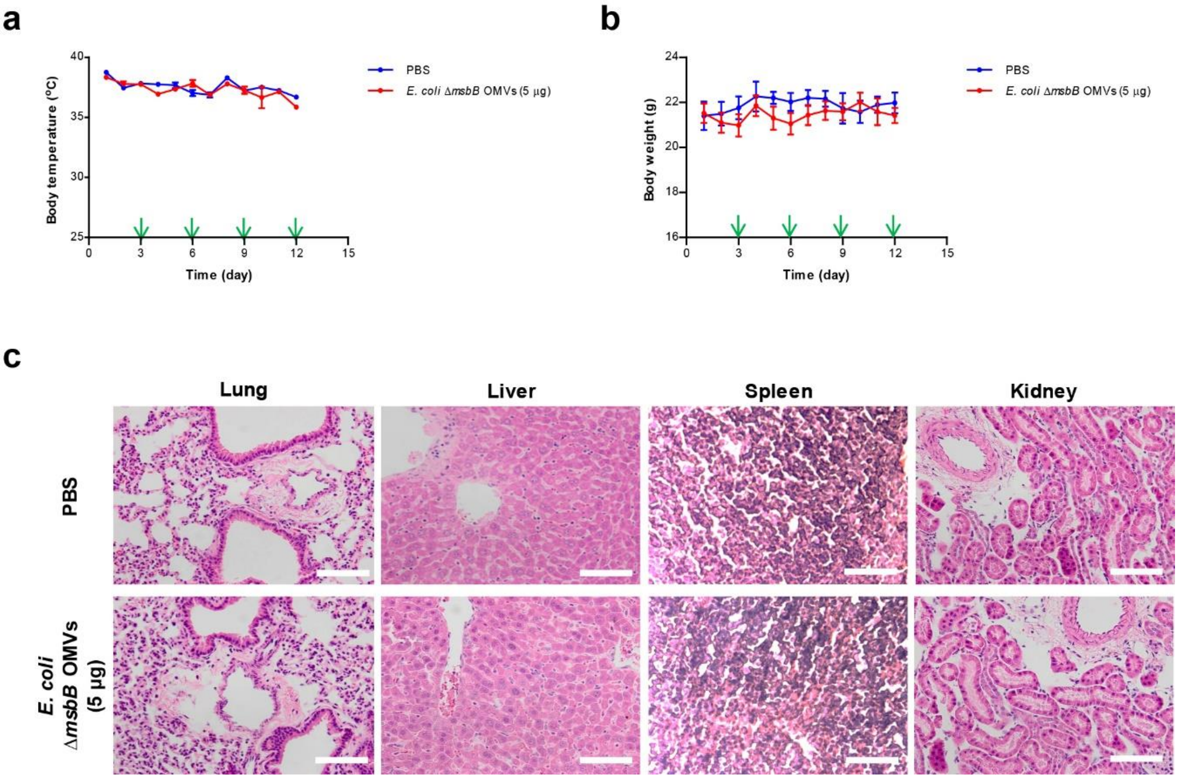

Supplementary Figure 3. Effect of *E. coli* Δ*msbB* OMV-treatments on body weight and temperature and organ histology. a,b Body temperature (**a**) and body weight (**b**) of mice bearing CT26 tumor measured after intravenous treatments of 5 µg *E. coli* Δ*msbB* OMVs (*n* = 7 mice per group). Green arrows indicate OMV-treatments. Data are presented as the mean ± s.d. from a representative experiments. **c,** Histology of the lung, liver, spleen and kidney extracted from the mice at day 15. Scale bars, 50 µm.

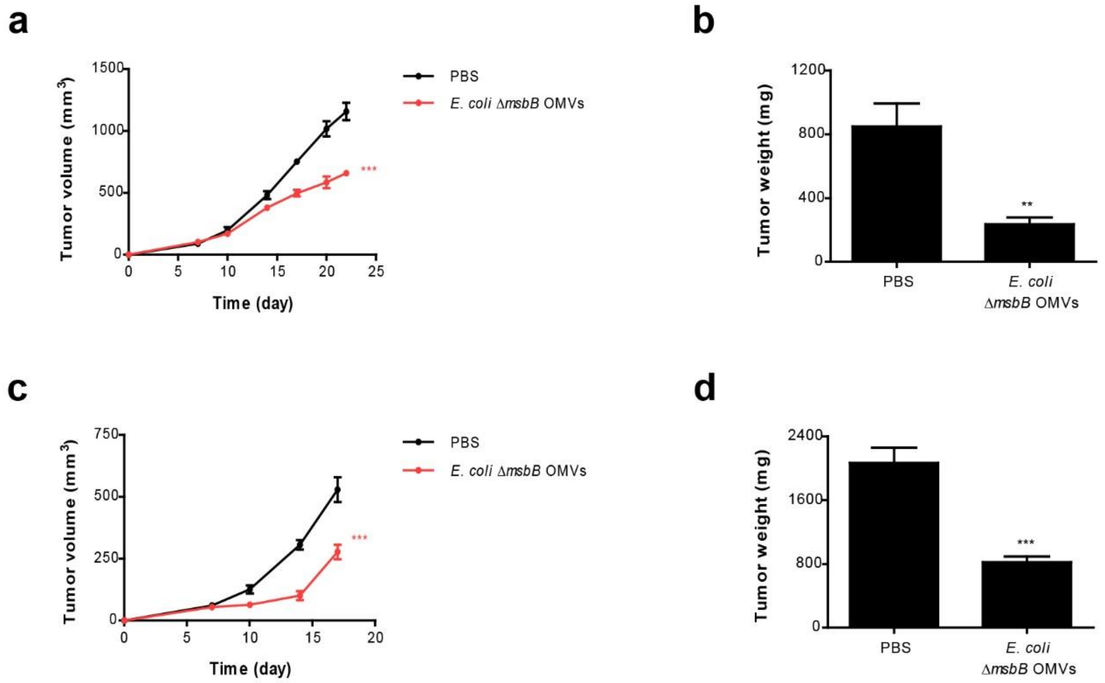

Supplementary Figure 4. Antitumor activity of *E. coli* Δ*msbB* OMVs on the primary tumor growth of highly metastatic 4T1 carcinoma and B16BL6 melanoma cells. Highly metastatic 4T1 carcinoma cells and B16BL6 melanoma cells were subcutaneously injected to the right flank of the mice (total $n = 12$ mice per group, two independent experiments). *E. coli* Δ*msbB* OMVs (5 μg) were injected intravenously four times from day 7 with 3 days interval. **a-d,** Primary tumor volume of mice bearing 4T1 (**a**) and B16BL6 (**c**) tumor measured after *E. coli* Δ*msbB* OMV-treatments. Weight of 4T1 (**b**) and B16BL6 (**d**) primary tumor of mice at the end of the experiment. ** and *** indicate $P < 0.01$ and $P < 0.001$, respectively, analysed by two-way ANOVA (**a, c**) and unpaired Student's t-test (**b, d**). Data are presented as the mean ± s.d. from a representative experiments.

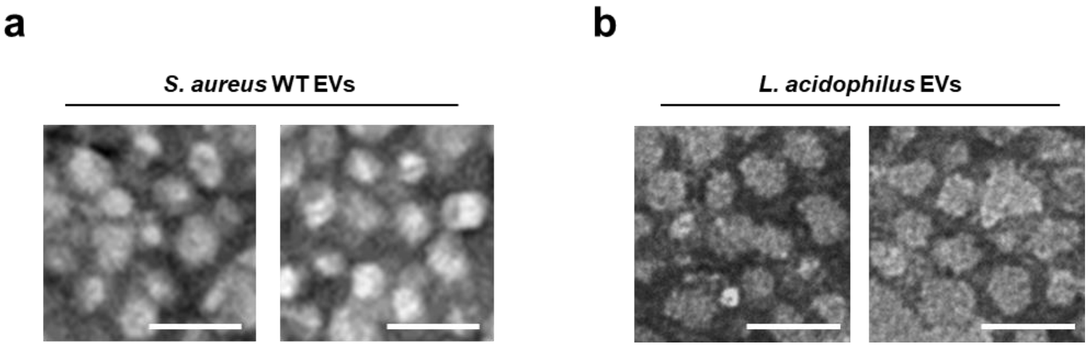

Supplementary Figure 5. **Transmission electron micrograph images of Gram-positive bacterial extracellular vesicles. a,b,** Transmission electron micrograph image of *S. aureus* wildtype (**a**) and *L. acidophilus* (**b**) extracellular vesicles. Scale bars, 50 nm.

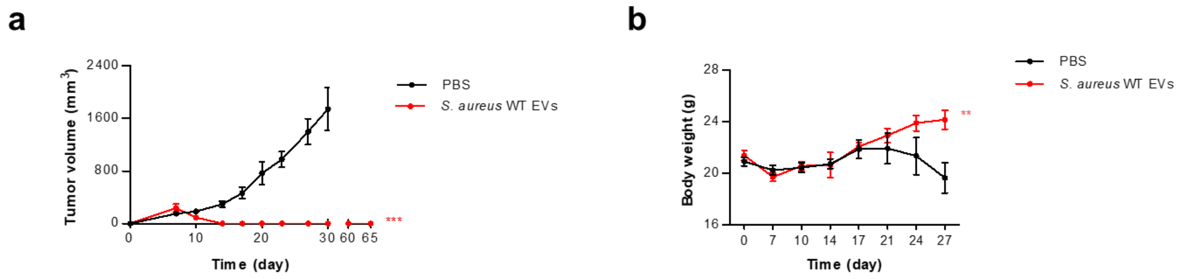

Supplementary Figure 6. Antitumor activity of Gram-positive *S. aureus* wildtype extracellular vesicles on CT26 tumor cells. To check if the tumor regression effect is maintained for long-term without tumor rebound, *S. aureus* wildtype extracellular vesicles (EVs) (5 μg in total protein amount) were injected intravenously four times from day 7 with 3 days intervals to BALB/c mice subcutaneously injected with CT26 tumor cells (total $n = 12$ mice per group, two independent experiments). **a,** Tumor volume measured after *S. aureus* extracellular vesicle-treatments. **b,** Body weights of mice measured after different time points after tumor challenge. ** and *** indicate $P < 0.01$ and $P < 0.001$, respectively, analysed by two-way ANOVA. Data are presented as the mean ± s.d. from a representative experiments.

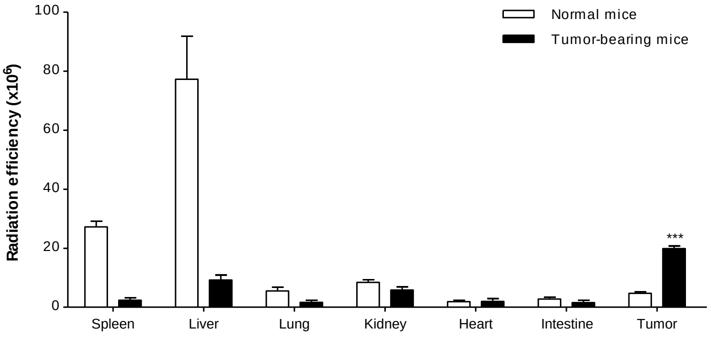

Supplementary Figure 7. **Targeting of *E. coli* Δ*msbB* OMVs to tumor tissues after systemic administration.** Cy7-labeled *E. coli* Δ*msbB* OMVs (Δ*msbB* ^{Cy7}OMVs) were systematically injected to BALB/c mice bearing CT26 tumor cells. For control, Δ*msbB* ^{Cy7}OMVs were also injected to healthy BALB/c mice with no tumor. Spleen, liver, kidney, lung, heart, intestine, and tumor tissues were isolated to measure the accumulation of Cy7 fluorescence in different organs. Radiant efficiencies of each organ were acquired for Cy7 fluorescence using Living Image 3.1 Software. Results are from three independent experiments (total n = 9). *** indicates $P < 0.001$ analysed by unpaired Student's t-test. Data are presented as the mean ± s.d. from a representative experiments.

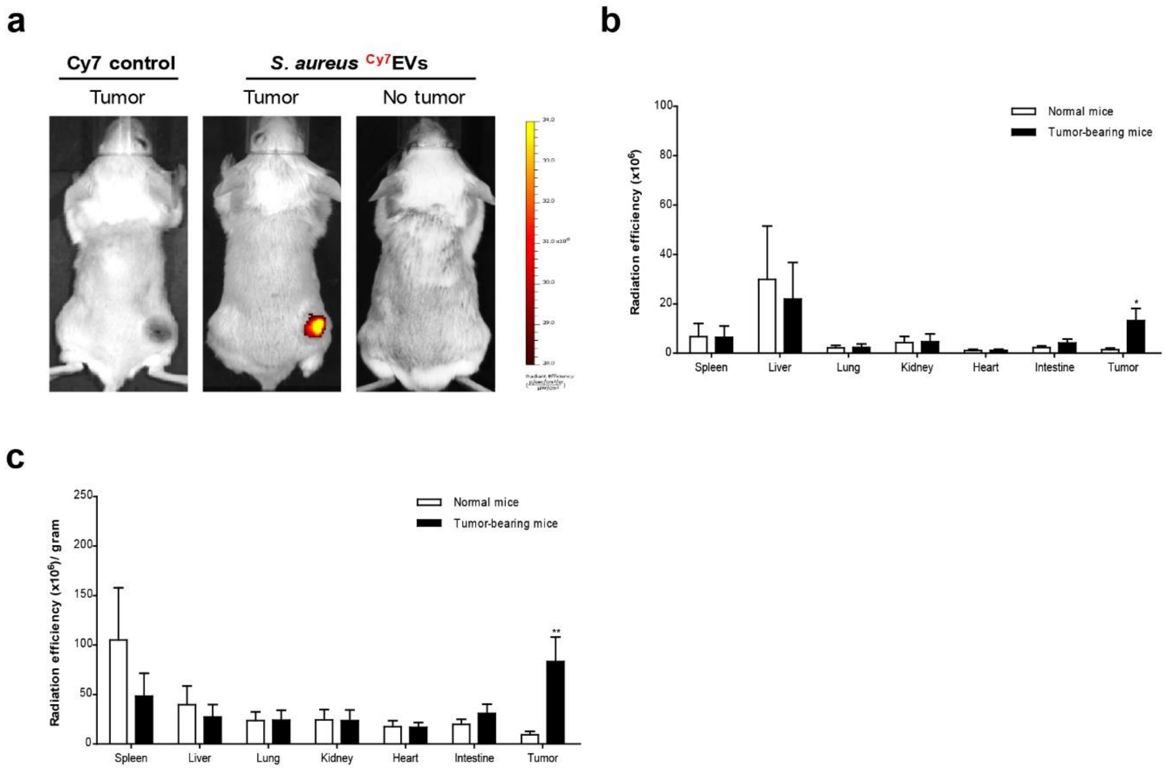

Supplementary Figure 8. **Targeting of *S. aureus* extracellular vesicles to tumor tissues after systemic administration. a,** Cy7 control and Cy7-labeled *S. aureus* wildtype extracellular vesicles (*S. aureus* ^{Cy7}EVs) were systematically injected to BALB/c mice bearing CT26 tumor cells. For control, *S. aureus* ^{Cy7}EVs were also injected to healthy BALB/c mice with no tumor. Whole body distributions of the injected ^{Cy7}EVs were observed using *in vivo* imaging system spectrum 12 h after the injection. **b,c,** To measure the accumulation of Cy7 fluorescence in different organs, spleen, liver, kidney, lung, heart, intestine, and tumor tissues were isolated. Radiant efficiencies of each organ were acquired for Cy7 fluorescence using Living Image 3.1 Software (**b**) and were normalized by each organ weight (**c**). Results are from three independent experiments (total n = 9). *, and ** indicate $P < 0.05$ and $P < 0.01$, respectively, analysed by unpaired Student's t-test. Data are presented as the mean ± s.d. from a representative experiments.

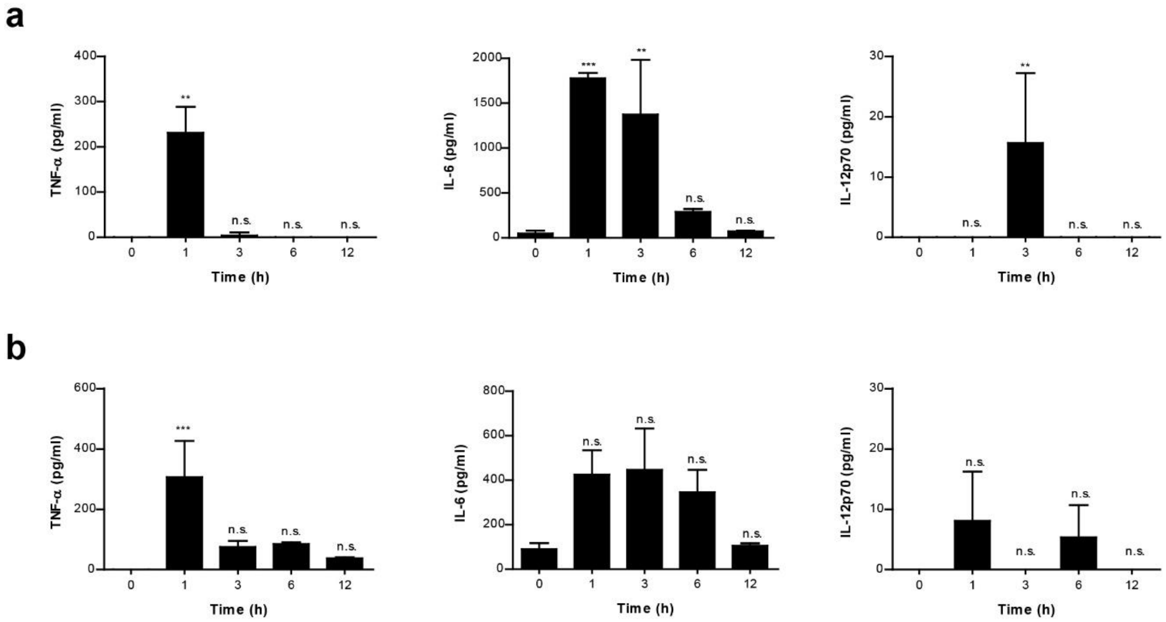

Supplementary Figure 9. **Kinetic quantification of cytokines after *E. coli* Δ*msbB* OMV injection. a,b,** Release of pro-inflammatory cytokines TNF-α, IL-6**,** and IL-12p70 in blood sera (**a**) and tumor cell lysates (**b**) after single intravenous injection of *E. coli* Δ*msbB* OMVs (5 μg in total protein) to mice bearing CT26 tumors at different time points. Data are shown as the mean ± s.d. (n = 6 mice per group). n.s., **, and *** indicate not significant, $P < 0.01$, and $P < 0.001$, respectively, analysed by one-way ANOVA with Bonferroni multiple comparisons post-test.

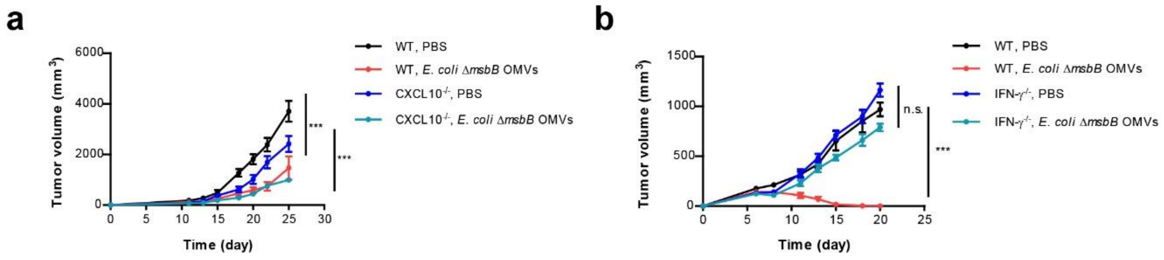

Supplementary Figure 10. **Effect of CXCL10 and IFN-γ on *E. coli* Δ*msbB* OMV antitumor response. a,** Tumor volume of wildtype C57BL/6 mice and CXCL10 knockout (CXCL10$^{-/-}$) mice bearing B16BL6 tumor measured after *E. coli* Δ*msbB* OMV-treatments. **b,** Tumor volume of wildtype BALB/c mice and IFN-γ knockout (IFN-γ$^{-/-}$) mice bearing CT26 tumor measured after *E. coli* Δ*msbB* OMV-treatments. Data are presented as the mean ± s.d. (n = 6 mice per group). n.s. and *** indicate non significant and $P < 0.001$, respectively, analysed by two-way ANOVA with Bonferroni multiple comparisons post-test.

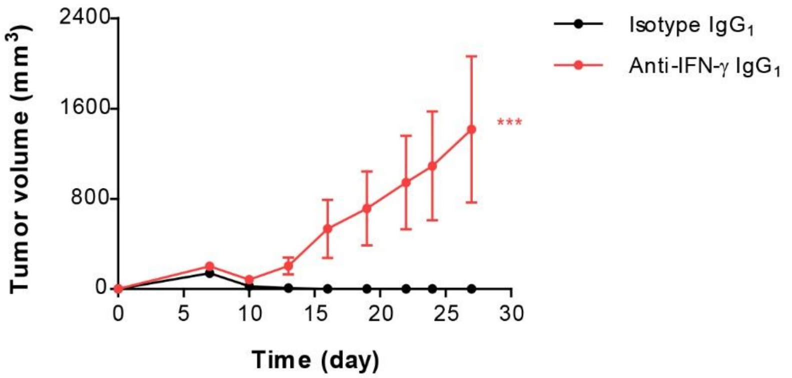

Supplementary Figure 11. Effect of IFN-γ on *E. coli* Δ*msbB* OMV antitumor response. Tumor volume of BALB/c mice bearing CT26 tumor measured after mouse monoclonal anti-IFN-γ antibodies (200 μg) and Δ*msbB* OMV (5 μg in total protein amount) treatments or control isotype IgG_1 antibodies (200 μg) and Δ*msbB* OMV (5 μg in total protein amount) treatments. Data are presented as the mean ± s.d. (n = 5 mice per group). *** indicates $P < 0.001$ analysed by two-way ANOVA.

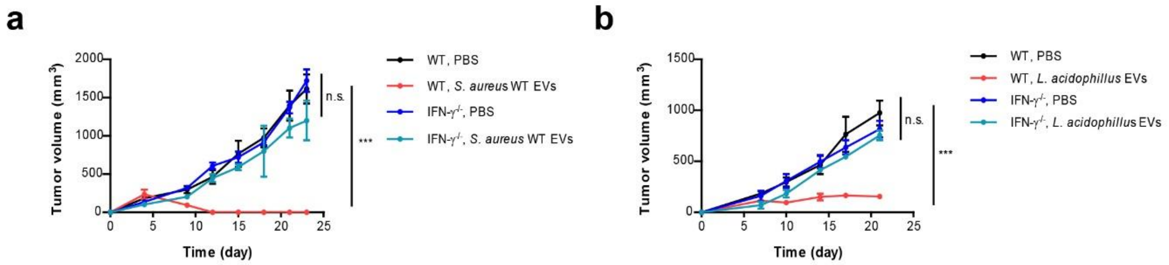

Supplementary Figure 12. **Effect of IFN-γ on Gram-positive bacterial extracellular vesicles antitumor response. a,b,** Tumor volume of wildtype BALB/c mice and IFN-γ-deficient (IFN-$\gamma^{-/-}$) mice bearing CT26 tumor measured after *S. aureus* wildtype extracellular vesicles (EVs) (**a**) and *L. acidophilus* extracellular vesicles (EVs) (**b**) treatments. Data are presented as the mean ± s.d. (n = 5 mice per group). n.s. and *** indicate non significant and $P < 0.001$, respectively, analysed by two-way ANOVA with Bonferroni multiple comparisons post-test.

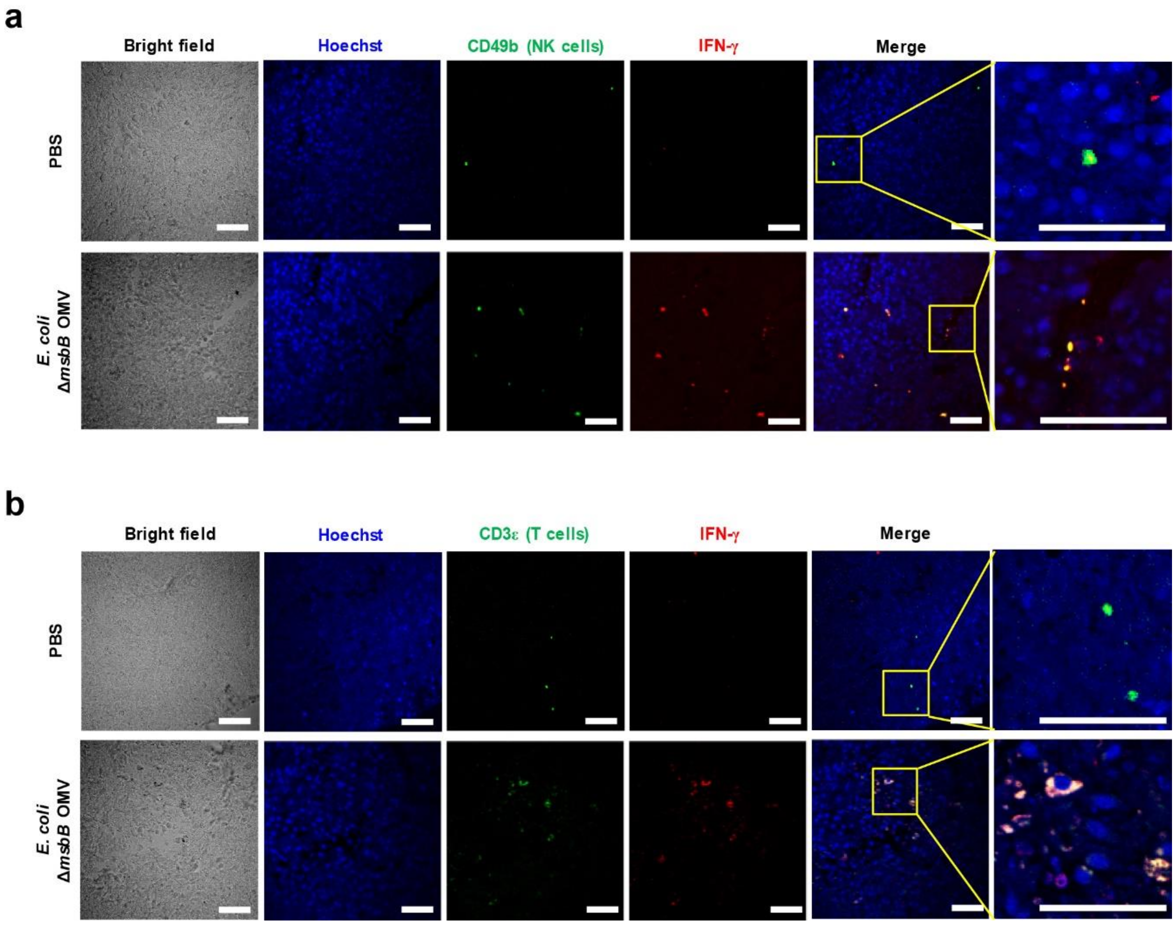

Supplementary Figure 13. NK and T cells produce IFN-γ in *E. coli* Δ*msbB* OMV-treated tumors. a,b,Confocal images of tumor tissues isolated from wildtype mice bearing CT26 tumors, stained for IFN-γ and NK cells (**a**) or T cells (**b**) 48 h after intravenous injections of *E. coli* ΔmsbB OMVs. The cell nucleus is stained in blue (Hoechst) while NK and T cells are shown by green fluorescence signal and IFN-γ is shown in red fluorescence signal, respectively. Scale bars, 50 μm.

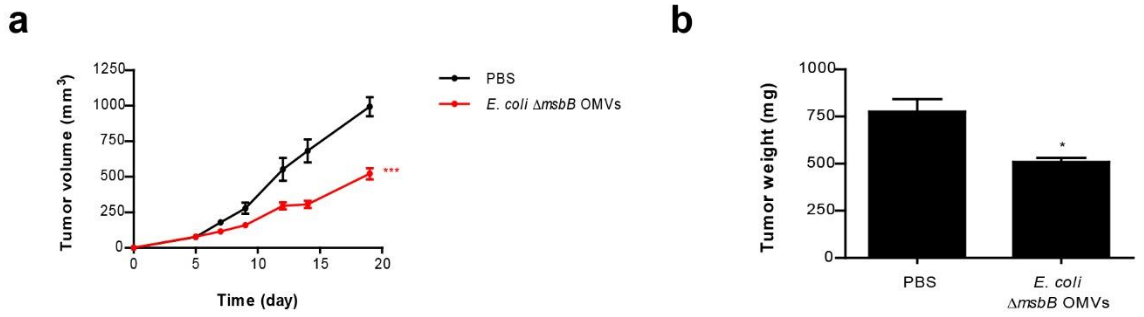

Supplementary Figure 14. **Importance of T cells on OMV antitumor effect. a,b,** Tumor volume of athymic nude (NU/J (Foxn1nu/Foxn1nu), F120) mice bearing CT26 tumor measured after *E. coli* Δ*msbB* OMV-treatments (**a**) and tumor weight at the end of the experiment (**b**). Data are presented as the mean ± s.d. (n = 6 mice per group). * and *** indicate $P < 0.05$ and $P < 0.001$, respectively, analysed by two-way ANOVA (**a**) and unpaired Student's t-test (**b**).

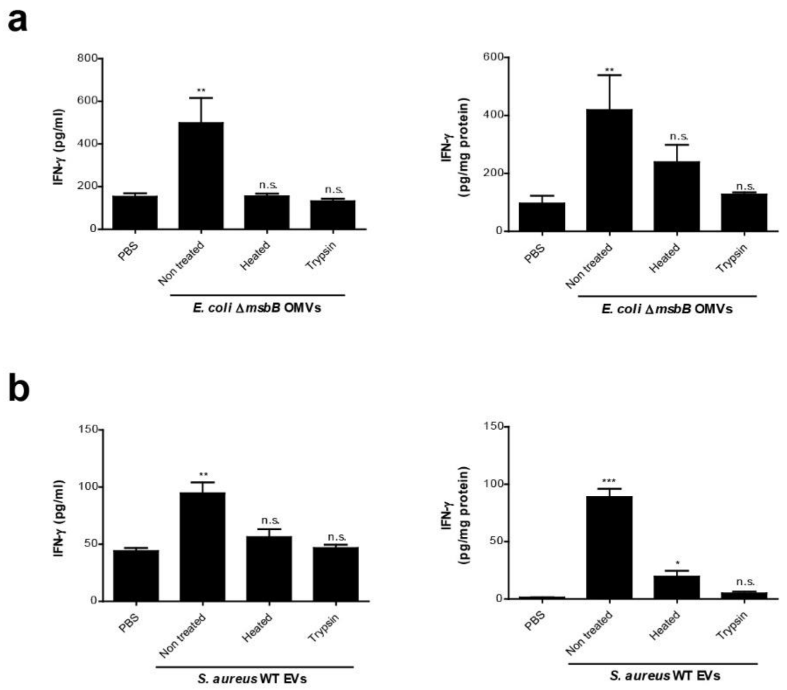

Supplementary Figure 15. Importance of vesicular surface proteins on IFN-γ production. **a,** Release of IFN-γ in blood sera at 6 h (left) and tumor cell lysates at 48 h (right) after single intravenous injection of intact, heated, or trypsin-treated *E. coli* Δ*msbB* OMVs (5 μg in total protein amount) to mice bearing CT26 tumors. **b,** Release of IFN-γ in blood sera at 6 h (left) and tumor cell lysates at 48 h (right) after single intravenous injection of intact, heated, or trypsin-treated *S. aureus* wildtype extracellular vesicles (EVs) (5 μg in total protein amount) to mice bearing CT26 tumors. Data are shown as the mean ± s.d. (n = 6 mice per group). n.s., *, **, and *** indicate not significant, $P < 0.05$, $P < 0.01$, and $P < 0.001$, respectively, analysed by one-way ANOVA. Bonferroni multiple comparisons post-test was applied to compare treated group with PBS group of each mouse.

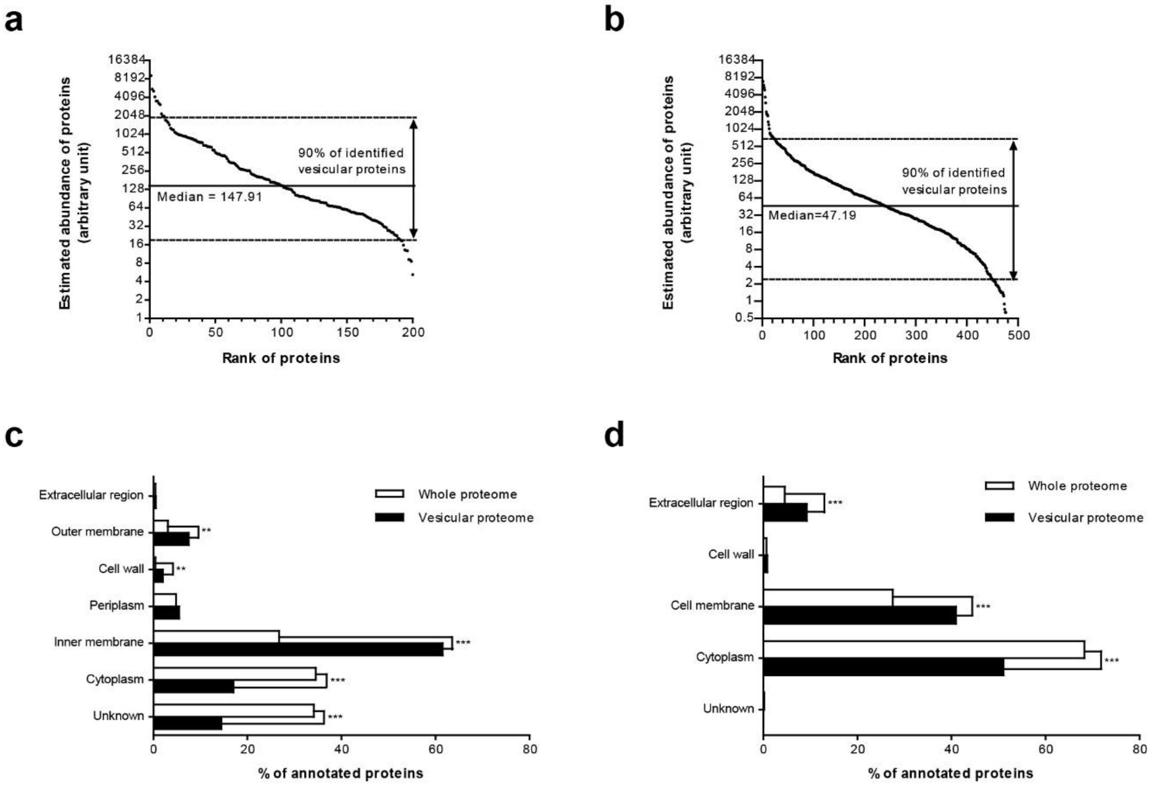

Supplementary Figure 16. Proteomic analysis on extracellular vesicles derived from *E. coli* W3110 *ΔmsbB* and wildtype *S. aureus*. a,b, The distribution of estimated abundance of *E. coli* W3110 *ΔmsbB* (**a**) and wildtype *S. aureus* (**b**) vesicular proteins according to their abundance ranks. Note that the most of the identified proteins (~90%) are located within a range from 19.70 to 2010.38 (**a**; median = 147.91), and from 2.25 to 696.12 (**b**; median = 47.19). Neither yeast nor cow proteins were identified from proteomic analyses on two types of extracellular vesicles. **c,d,** Enriched subcellular localizations of *E. coli* W3110 *ΔmsbB* (**c**) and wildtype *S. aureus* (**d**) vesicular proteins over the cellular proteins. White and black bars indicate the percentages in cellular and vesicular proteins, respectively. ** and *** indicate $P < 0.01$ and $P < 0.001$, respectively, analysed by Fisher's exact test.

Supplementary Table 1. Proteins identified from *E. coli* W3110 *ΔmsbB* outer membrane vesicles (OMVs)

Index	Protein name	UniProt accession	Gene symbol	APEX score[a)]
Extracellular region				
1	Flagellin	P04949	fliC	82.40
Outer membrane				
1	Outer membrane protein C	P06996	ompC	260.67
2	Outer membrane protein A	P0A910	ompA	112.98
3	Protease 7	P09169	ompT	84.13
4	Probable outer membrane usher protein ElfC	P75857	elfC	69.65
5	Outer membrane protein X	P0A917	ompX	66.31
6	Putative outer membrane usher protein YfcU	P77196	yfcU	61.85
7	Membrane-bound lytic murein transglycosylase A	P0A935	mltA	61.14
8	Outer-membrane lipoprotein LolB	P61320	lolB	42.43
9	Probable lipoprotein YiaD	P37665	yiaD	40.24
10	Maltoporin	P02943	lamB	39.16
11	Nucleoside-specific channel-forming protein tsx	P0A927	tsx	36.37
12	Membrane-bound lytic murein transglycosylase F	P0AGC5	mltF	27.32
13	Outer membrane protein assembly factor BamA	P0A940	bamA	26.44
14	Peptidoglycan-associated lipoprotein	P0A912	pal	18.71
15	Vitamin B12 transporter BtuB	P06129	btuB	5.19
Cell wall				
1	Penicillin-binding protein 1B	P02919	mrcB	752.68
2	Cytochrome c-type protein TorC	P33226	torC	264.37
3	Cytochrome c-type protein TorY	P52005	torY	92.92
4	Bifunctional (p)ppGpp synthase/hydrolase SpoT	P0AG24	spoT	20.39
Periplasm				
1	Acid stress chaperone HdeA	P0AES9	hdeA	1036.60
2	Protein TonB	P02929	tonB	900.91
3	Zinc resistance-associated protein	P0AAA9	zraP	817.37
4	Blue copper oxidase CueO	P36649	cueO	484.20
5	Thiol:disulfide interchange protein DsbE	P0AA86	dsbE	333.13
6	Thiol:disulfide interchange protein DsbG	P77202	dsbG	67.27
7	Formate-dependent nitrite reductase complex subunit NrfF	P32711	nrfF	64.54
8	Uncharacterized ferredoxin-like protein YdhX	P77375	ydhX	60.08
9	Probable L,D-transpeptidase YbiS	P0AAX8	ybiS	50.72
10	Protein TolB	P0A855	tolB	12.77
11	Maltose-binding periplasmic protein	P0AEX9	malE	8.55
Inner membrane				
1	Endolytic murein transglycosylase	P28306	mltG	3559.64
2	Hydrogenase-4 component F	P77437	hyfF	3259.59
3	Uncharacterized permease YjhF	P39357	yjhF	3104.58
4	Flagellar motor switch protein FliN	P15070	fliN	2191.00
5	Inner membrane protein YdgC	P0ACX0	ydgC	1739.67
6	Arginine ABC transporter permease protein ArtM	P0AE30	artM	1678.92
7	Phosphatidylglycerophosphatase C	P0AD42	pgpC	1589.05
8	Small toxic protein TisB	A5A627	tisB	1452.02
9	Cell division protein ZipA	P77173	zipA	1274.20

10	Uncharacterized protein YggT	P64564	yggT	1230.03
11	Inner membrane protein YcfZ	P75961	ycfZ	1210.64
12	ATP synthase protein I	P0ABC0	atpI	1066.52
13	Uncharacterized protein YigF	P27842	yigF	1014.01
14	Ferric enterobactin transport system permease protein FepD	P23876	fepD	990.28
15	Probable acyltransferase YihG	P32129	yihG	985.51
16	Protein SanA	P0AFY2	sanA	962.04
17	Inner membrane protein YnbA	P76090	ynbA	947.46
18	Inner membrane protein YbaN	P0AAR5	ybaN	945.14
19	Glycine betaine uptake system permease protein YehY	P33361	yehY	926.27
20	Protein TonB	P02929	tonB	900.91
21	Multidrug export protein EmrA	P27303	emrA	883.58
22	Flagellar biosynthetic protein FliQ	P0AC07	fliQ	858.53
23	Cytochrome bo(3) ubiquinol oxidase subunit 2	P0ABJ1	cyoA	855.62
24	High-affinity gluconate transporter	P39835	gntT	781.63
25	Phage shock protein G	P32696	pspG	760.02
26	Penicillin-binding protein 1B	P02919	mrcB	752.68
27	Uncharacterized inner membrane transporter YedA	P0AA70	yedA	744.65
28	Uncharacterized membrane protein YohP	C1P609	yohP	674.15
29	Glutathione-regulated potassium-efflux system protein KefB	P45522	kefB	669.47
30	ATP synthase subunit c	P68699	atpE	661.73
31	UPF0057 membrane protein YqaE	P0AE42	yqaE	643.68
32	Enterobactin exporter EntS	P24077	entS	635.07
33	Multidrug resistance protein MdtB	P76398	mdtB	630.64
34	2-keto-3-deoxygluconate permease	P0A712	kdgT	626.00
35	Inner membrane protein YghQ	Q46841	yghQ	565.42
36	DsdX permease	P08555	dsdX	558.86
37	Nickel transport system permease protein NikC	P0AFA9	nikC	518.43
38	Putative electron transport protein YccM	P52636	yccM	494.15
39	Glutathione-regulated potassium-efflux system protein KefC	P03819	kefC	464.60
40	Sec-independent protein translocase protein TatA	P69428	tatA	455.76
41	Ferredoxin-type protein NapH	P33934	napH	455.50
42	Lipid A export ATP-binding/permease protein MsbA	P60752	msbA	453.01
43	Tyrosine-protein kinase wzc	P76387	wzc	406.08
44	Inner membrane protein YphA	P0AD47	yphA	370.29
45	Nitrate/nitrite sensor protein NarX	P0AFA2	narX	352.46
46	Phosphate transport system permease protein PstC	P0AGH8	pstC	350.56
47	Peptide transport system permease protein SapB	P0AGH3	sapB	347.35
48	Anaerobic C4-dicarboxylate transporter DcuA	P0ABN5	dcuA	343.69
49	Thiol:disulfide interchange protein DsbE	P0AA86	dsbE	333.13
50	Signal transduction histidine-protein kinase AtoS	Q06067	atoS	302.21
51	Uncharacterized protein YdbH	P52645	ydbH	283.35
52	Probable cystine transporter YijE	P0ABT8	yijE	273.33
53	Cation/acetate symporter ActP	P32705	actP	269.66
54	Cytochrome c-type protein TorC	P33226	torC	264.37
55	Probable Ni/Fe-hydrogenase 2 b-type cytochrome subunit	P37180	hybB	258.98
56	Protein DedA	P0ABP6	dedA	258.11
57	Low conductance mechanosensitive channel YnaI	P0AEB5	ynaI	235.59
58	Protein sirB2	Q46755	ychQ	222.09
59	Low-affinity gluconate transporter	P0AC96	gntU	215.17
60	Protein HemY	P0ACB7	hemY	206.16
61	Inner membrane protein YgiZ	Q46867	ygiZ	202.47

62	UPF0259 membrane protein YciC	P21365	yciC	201.70
63	Arsenical pump membrane protein	P0AB93	arsB	198.73
64	Sec-independent protein translocase protein TatE	P0A843	tatE	189.49
65	Formate dehydrogenase, nitrate-inducible, iron-sulfur subunit	P0AAJ3	fdnH	185.89
66	Putrescine transport system permease protein PotH	P31135	potH	185.75
67	L-fucose-proton symporter	P11551	fucP	182.44
68	Flagellar protein FliO	P22586	fliO	167.98
69	Iron(3+)-hydroxamate import system permease protein FhuB	P06972	fhuB	165.98
70	Cell shape-determining protein MreC	P16926	mreC	159.45
71	Nucleoside permease NupC	P0AFF2	nupC	136.94
72	Inner membrane ABC transporter permease protein YcjO	P0AFR7	ycjO	132.69
73	Formate hydrogenlyase subunit 4	P16430	hycD	129.49
74	Sodium/proline symporter	P07117	putP	125.31
75	Fosmidomycin resistance protein	P52067	fsr	116.82
76	Inner membrane transport permease YbhR	P0AFP9	ybhR	102.52
77	Sensor protein PhoQ	P23837	phoQ	98.20
78	Formate dehydrogenase-O iron-sulfur subunit	P0AAJ5	fdoH	97.65
79	Inner membrane protein YjiY	P39396	yjiY	97.58
80	Ribosome-associated ATPase	P37624	rbbA	95.20
81	Cytochrome c-type protein TorY	P52005	torY	92.92
82	DNA translocase FtsK	P46889	ftsK	92.85
83	PTS system N-acetylglucosamine-specific EIICBA component	P09323	nagE	87.16
84	UPF0092 membrane protein YajC	P0ADZ7	yajC	86.41
85	High-affinity branched-chain amino acid transport system permease protein LivM	P22729	livM	84.91
86	Proton/glutamate-aspartate symporter	P21345	gltP	84.07
87	Uncharacterized protein YcgG	P75995	ycgG	82.85
88	Flagellin	P04949	fliC	82.40
89	Elongation factor Tu 1	P0CE47	tufA	78.42
90	O-antigen ligase	P27243	rfaL	78.03
91	Modulator of FtsH protease HflC	P0ABC3	hflC	77.38
92	Putative transport protein YidE	P60872	yidE	77.12
93	Cyclic di-GMP-binding protein	P37652	bcsB	71.78
94	Cell division protein DamX	P11557	damX	69.14
95	DNA-damage-inducible protein F	P28303	dinF	68.54
96	Succinate dehydrogenase iron-sulfur subunit	P07014	sdhB	68.33
97	Inner membrane protein YeeR	P76361	yeeR	65.11
98	Membrane-bound lytic murein transglycosylase A	P0A935	mltA	61.14
99	Thiamine transport system permease protein ThiP	P31549	thiP	60.00
100	Uracil permease	P0AGM7	uraA	57.89
101	Cell division protein DedD	P09549	dedD	55.03
102	Small-conductance mechanosensitive channel	P0C0S1	mscS	54.24
103	Inner membrane protein YabI	P30149	yabI	54.13
104	Protein AbrB	P75747	abrB	52.15
105	Inner membrane ABC transporter ATP-binding protein YddA	P31826	yddA	50.95
106	Uncharacterized transporter YeaV	P0ABD1	yeaV	50.60
107	Putative O-antigen transporter	P37746	rfbX	49.21
108	Dihydrolipoyl dehydrogenase	P0A9P0	lpdA	48.02
109	Guanine/hypoxanthine permease GhxP	P0AF52	ghxP	46.14
110	Respiratory nitrate reductase 2 beta chain	P19318	narY	44.98
111	ATP synthase subunit alpha	P0ABB0	atpA	43.54
112	Probable lipoprotein YiaD	P37665	yiaD	40.24

113	Uncharacterized ABC transporter ATP-binding protein YejF	P33916	yejF	40.23
114	Lipid III flippase	P0AAA7	wzxE	34.07
115	Methyl-accepting chemotaxis protein II	P07017	tar	31.82
116	ATP synthase subunit beta	P0ABB4	atpD	27.33
117	Chain length determinant protein	P76372	wzzB	25.31
118	Uncharacterized protein YhgE	P45804	yhgE	22.34
119	Bifunctional (p)ppGpp synthase/hydrolase SpoT	P0AG24	spoT	20.39
120	RNA polymerase sigma factor RpoH	P0AGB3	rpoH	18.51
121	Mechanosensitive channel MscK	P77338	mscK	15.69
122	Inner membrane protein YccS	P75870	yccS	12.86
123	UPF0748 lipoprotein YddW	P64426	yddW	9.04
Cytoplasm				
1	FKBP-type peptidyl-prolyl cis-trans isomerase SlyD	P0A9K9	slyD	9250.87
2	Regulator of ribonuclease activity B	P0AF90	rraB	5193.47
3	Protein PhnA	P0AFJ1	phnA	4173.32
4	UPF0381 protein YfcZ	P0AD33	yfcZ	3553.43
5	30S ribosomal protein S6	P02358	rpsF	817.30
6	Dual-specificity RNA methyltransferase RlmN	P36979	rlmN	482.42
7	Sec-independent protein translocase protein TatA	P69428	tatA	455.76
8	GTPase Der	P0A6P5	der	315.82
9	RNA polymerase sigma factor RpoD	P00579	rpoD	274.95
10	Uncharacterized protein YicH	P31433	yicH	210.82
11	Stringent starvation protein B	P0AFZ3	sspB	192.56
12	Dihydroxy-acid dehydratase	P05791	ilvD	168.24
13	Thymidylate synthase	P0A884	thyA	161.47
14	GTPase ObgE/CgtA	P42641	obgE	137.47
15	tRNA-specific 2-thiouridylase MnmA	P25745	mnmA	98.75
16	Ribosome-associated ATPase	P37624	rbbA	95.20
17	Putative uncharacterized protein YehQ	P33353	yehQ	83.29
18	Uncharacterized protein YcgG	P75995	ycgG	82.85
19	Elongation factor Tu 1	P0CE47	tufA	78.42
20	Uncharacterized sugar kinase YgcE	P55138	ygcE	78.32
21	Membrane-bound lytic murein transglycosylase A	P0A935	mltA	61.14
22	Dihydrolipoyllysine-residue acetyltransferase component of pyruvate dehydrogenase complex	P06959	aceF	57.33
23	DNA-directed RNA polymerase subunit beta	P0A8V2	rpoB	53.40
24	60 kDa chaperonin	P0A6F5	groL	51.70
25	Malate dehydrogenase	P61889	mdh	51.57
26	Dihydrolipoyl dehydrogenase	P0A9P0	lpdA	48.02
27	HMP-PP phosphatase	P46891	cof	38.46
28	Pyruvate dehydrogenase E1 component	P0AFG8	aceE	35.32
29	30S ribosomal protein S7	P02359	rpsG	23.58
30	DNA-directed RNA polymerase subunit beta'	P0A8T7	rpoC	22.77
31	Bifunctional (p)ppGpp synthase/hydrolase SpoT	P0AG24	spoT	20.39
32	Lysine--tRNA ligase	P0A8N3	lysS	19.71
33	RNA polymerase sigma factor RpoH	P0AGB3	rpoH	18.51
34	Glutamine synthetase	P0A9C5	glnA	9.12
Unknown				
1	Uncharacterized protein YpjK	P52134	ypjK	5582.98
2	Uncharacterized protein YkfM	A5A605	ykfM	2014.71
3	Uncharacterized ferredoxin-like protein YfhL	P52102	yfhL	2010.38
4	Aldehyde reductase YahK	P75691	yahK	1102.66
5	Small toxic protein ShoB	C1P611	shoB	886.83

6	Uncharacterized protein YdaT	P76064	ydaT	748.32
7	Xanthine dehydrogenase iron-sulfur-binding subunit	Q46801	xdhC	436.09
8	UPF0153 protein YeiW	P0AFT8	yeiW	266.54
9	Uncharacterized protein YpjB	P76612	ypjB	223.76
10	Uncharacterized protein YhdP	P46474	yhdP	206.17
11	Antitermination protein Q homolog from lambdoid prophage DLP12	Q47274	quuD	166.92
12	Transposase InsD for insertion element IS2A	P0CF53	insD1	157.69
13	Uncharacterized protein DinQ	A5A624	dinQ	153.34
14	Uncharacterized protein YjfM	P39295	yjfM	147.92
15	Protein FixC	P68644	fixC	147.92
16	Uncharacterized protein YdfU	P76162	ydfU	133.09
17	Protein YdeP	P77561	ydeP	129.36
18	UPF0260 protein YcgN	P0A8L5	ycgN	103.02
19	Putative glycyl-radical enzyme activating enzyme YjjW	P39409	yjjW	101.00
20	Uncharacterized protein YkgJ	P0AAL9	ykgJ	91.46
21	IlvGMEDA operon leader peptide	P62522	ilvL	67.39
22	Exodeoxyribonuclease 8	P15032	recE	65.77
23	Putative cyclic di-GMP phosphodiesterase YliE	P75800	yliE	61.05
24	Alpha-xylosidase	P31434	yicI	40.72
25	Uncharacterized protein YccE	P36661	yccE	37.37
26	Sulfoquinovosidase	P32138	yihQ	31.27
27	Putative uncharacterized protein YjiV	P39393	yjiV	30.27
28	FeS cluster assembly protein SufB	P77522	sufB	25.37
29	GDP-mannose 4,6-dehydratase	P0AC88	gmd	13.10

[a)] The proteins are ordered from the highest to lowest APEX score. APEX normalization factor = 100,000.

Supplementary Table 2. Proteins identified from *S. aureus* RN4220 extracellular vesicles

Index	Protein name	UniProt accession	Gene symbol	APEX score[a)]
Extracellular region				
1	Staphylococcal secretory antigen ssaA2	Q2G2J2	ssaA2	4977.34
2	ABC transporter, substrate-binding protein, putative	Q2G2D8	SAOUHSC_00634	3754.05
3	5'-nucleotidase, lipoprotein e(P4) family	Q2G170	SAOUHSC_00284	2960.72
4	Bifunctional autolysin	Q2FZK7	atl	1757.82
5	N-acetylmuramoyl-L-alanine amidase sle1	Q2G0U9	sle1	794.68
6	ABC transporter periplasmic binding protein, putative	Q2FW75	SAOUHSC_02430	671.36
7	Lipoprotein	Q2FZZ0	SAOUHSC_00844	628.90
8	Uncharacterized protein	Q2G0F2	SAOUHSC_00617	545.59
9	Uncharacterized protein	Q2FVW9	SAOUHSC_02554	491.28
10	Lipase 2	Q2G155	lip2	490.60
11	Periplasmic binding protein, putative	Q2G1N4	SAOUHSC_00074	390.76
12	Uncharacterized protein	Q2G019	SAOUHSC_00808	378.24
13	Probable transglycosylase IsaA	Q2FV52	isaA	309.40
14	Enolase	Q2G028	eno	272.29
15	LysM domain protein	Q2FV81	SAOUHSC_02855	234.21
16	Uncharacterized protein	Q2G0Z9	SAOUHSC_00362	214.11
17	Uncharacterized protein	Q2G071	SAOUHSC_00749	204.08
18	Molybdenum ABC transporter, periplasmic molybdate-binding protein	Q2FVX4	SAOUHSC_02549	199.91
19	Uncharacterized protein	Q2G264	SAOUHSC_01180	188.47
20	Staphylococcal secretory antigen SsaA	Q2FV55	ssaA	180.57
21	Uncharacterized protein	Q2FVF5	SAOUHSC_02759	152.59
22	N-acetylmuramoyl-L-alanine amidase domain-containing protein SAOUHSC_02979	Q2G222	SAOUHSC_02979	131.51
23	Uncharacterized protein	Q2FZG5	SAOUHSC_01039	98.77
24	Immunoglobulin-binding protein sbi	Q2FVK5	sbi	83.10
25	Peptide ABC transporter, peptide-binding protein, putative	Q2FVE7	SAOUHSC_02767	56.71
26	Uncharacterized protein	Q2FWY6	SAOUHSC_02121	55.83
27	Lipase 1	Q2FUU5	lipA	55.27
28	Lipoprotein	Q2G0V0	SAOUHSC_00426	40.66
29	Uncharacterized protein	Q2FVL7	SAOUHSC_02694	36.93
30	Uncharacterized protein	Q2G0C0	SAOUHSC_00685	31.61
31	Uncharacterized protein	Q2FY39	SAOUHSC_01627	27.70
32	Uncharacterized protein	Q2G174	SAOUHSC_00279	24.90

33	Uncharacterized protein	Q2G2G0	SAOUHSC_00717	22.25
34	Probable cell wall amidase LytH	Q2FXU3	lytH	17.94
35	Uncharacterized protein	Q2G2Y5	SAOUHSC_01920	17.60
36	Uncharacterized protein SAOUHSC_00997	P52078	SAOUHSC_00997	14.79
37	Staphopain A	Q2G2R8	sspP	7.88
38	Bacterial extracellular solute-binding protein, putative	Q2G1E9	SAOUHSC_00176	7.51
39	Serine-aspartate repeat-containing protein D	Q2G0L4	sdrD	6.70
40	Iron-regulated surface determinant protein A	Q2FZE9	isdA	6.11
41	Serine-rich adhesin for platelets	Q2FUW1	sraP	5.01
42	Lipoteichoic acid synthase	Q2G093	ltaS	4.75
43	Uncharacterized protein	Q2G2T0	SAOUHSC_01969	4.06
44	Uncharacterized protein	Q2FWN6	SAOUHSC_02246	1.86

Cell wall

1	Uncharacterized protein	Q2G242	SAOUHSC_00508	53.35
2	Serine-aspartate repeat-containing protein D	Q2G0L4	sdrD	6.70
3	Iron-regulated surface determinant protein A	Q2FZE9	isdA	6.11
4	Serine-rich adhesin for platelets	Q2FUW1	sraP	5.01

Cell membrane

1	Uncharacterized protein	Q2FYZ6	SAOUHSC_01275	882.43
2	ATP synthase subunit beta	Q2FWF0	atpD	830.88
3	UPF0365 protein SAOUHSC_01676	Q2FXZ9	SAOUHSC_01676	712.09
4	ATP synthase subunit alpha	Q2FWE8	atpA	696.11
5	Succinate dehydrogenase, flavoprotein chain TC0881, putative	Q2FZC8	SAOUHSC_01104	685.62
6	UPF0154 protein SAOUHSC_01338	Q2FYT7	SAOUHSC_01338	590.02
7	ATP synthase subunit b	Q2G2F8	atpF	549.54
8	Probable quinol oxidase subunit 2	Q2FZJ9	qoxA	512.45
9	Preprotein translocase, YajC subunit	Q2FXT7	SAOUHSC_01747	430.95
10	UPF0478 protein SAOUHSC_01855	Q2G247	SAOUHSC_01855	392.78
11	Signal peptidase I	Q2FZT7	SAOUHSC_00903	371.46
12	Probable quinol oxidase subunit 1	Q2FZK0	qoxB	364.66
13	PTS system glucose-specific EIICBA component	Q2G1G8	ptsG	351.98
14	Penicillin-binding protein 2	Q2FYI0	SAOUHSC_01467	318.86
15	Uncharacterized protein	Q2FXJ6	SAOUHSC_01838	305.99
16	Uncharacterized protein	Q2G193	SAOUHSC_00253	299.58
17	Uncharacterized lipoprotein SAOUHSC_02650	Q2FVQ2	SAOUHSC_02650	288.26

18	Uncharacterized protein	Q2G0W5	SAOUHSC_00409	287.89
19	Uncharacterized protein	Q2G1W5	SAOUHSC_01908	286.75
20	Foldase protein PrsA	Q2G2S6	prsA	285.64
21	Uncharacterized protein	Q2FVL2	SAOUHSC_02699	280.26
22	Cell division protein FtsA	O07325	ftsA	274.81
23	Uncharacterized protein	Q2G257	SAOUHSC_01477	266.27
24	Iron-sulphur subunit of succinate dehydrogenase, putative	Q2FZC7	SAOUHSC_01105	262.13
25	MHC class II analog protein	Q2FWW1	SAOUHSC_02161	261.04
26	Uncharacterized protein	Q2FXG3	SAOUHSC_01884	239.39
27	ATP synthase gamma chain	Q2FWE9	atpG	208.48
28	Phospho-N-acetylmuramoyl-pentapeptide-transferase	Q2FZ93	mraY	198.19
29	PTS system, fructose-specific IIABC component, putative	Q2FUX0	SAOUHSC_02975	187.12
30	Uncharacterized protein	Q2FWX6	SAOUHSC_02145	183.08
31	Septation ring formation regulator EzrA	Q2FXK8	ezrA	175.50
32	Transcriptional regulator, putative	Q2FVV8	SAOUHSC_02583	168.04
33	L-lactate permease	Q2G1N9	SAOUHSC_00067	165.74
34	Uncharacterized protein	Q2FVN6	SAOUHSC_02666	162.55
35	Uncharacterized protein	Q2G0U0	SAOUHSC_00437	159.75
36	Sodium/proline symporter	Q2FWY7	putP	156.54
37	ATP-dependent zinc metalloprotease FtsH	Q2G0R0	ftsH	150.67
38	Extracellular matrix protein-binding protein emp	Q2G012	emp	149.93
39	Extramembranal protein	Q2FZW3	SAOUHSC_00872	148.89
40	Uncharacterized protein	Q2G082	SAOUHSC_00738	144.94
41	UPF0316 protein SAOUHSC_02131	Q2G2R9	SAOUHSC_02131	144.40
42	Glycine betaine transporter, putative	Q2FYT0	SAOUHSC_01346	142.23
43	Uncharacterized protein	Q2G2W5	SAOUHSC_02630	139.00
44	Uncharacterized protein	Q2FY36	SAOUHSC_01630	133.75
45	Uncharacterized protein	Q2FWX0	SAOUHSC_02151	129.50
46	Uncharacterized protein	Q2FVB9	SAOUHSC_02815	122.39
47	Na+/H+ antiporter, putative	Q2FVU1	SAOUHSC_02601	118.89
48	Glycerol-3-phosphate acyltransferase	Q2FYS6	plsY	117.74
49	TagB protein, putative	Q2G1C2	tarK	113.97
50	Uncharacterized protein	Q2FY58	SAOUHSC_01607	111.91
51	Sortase, putative	Q2FV99	SAOUHSC_02834	109.85
52	Membrane protein insertase YidC	Q2FWG4	yidC	107.03
53	Uncharacterized lipoprotein SAOUHSC_00405	Q2G0W9	SAOUHSC_00405	104.95
54	Lytic regulatory protein, putative	Q2FWA8	SAOUHSC_02390	104.53

55	Elastin-binding protein EbpS	Q2FYF1	ebpS	104.44
56	Protein translocase subunit SecY	O08387	secY	103.90
57	Protein-export membrane protein SecDF	Q2FXT8	secD	100.42
58	Uncharacterized protein	Q2FYQ3	SAOUHSC_01382	97.51
59	Sodium:alanine symporter family protein, putative	Q2G2N5	SAOUHSC_01354	96.54
60	Monofunctional glycosyltransferase	Q93Q23	mgt	93.32
61	PTS system EIIBC component SAOUHSC_00158	Q2G1G5	SAOUHSC_00158	93.07
62	Large-conductance mechanosensitive channel	P68805	mscL	92.70
63	Uncharacterized protein	Q2FXE7	SAOUHSC_01902	90.89
64	Sodium-dependent dicarboxylate transporter SdcS	Q2FWY4	sdcS	89.97
65	O-acetyltransferase OatA	Q2FV54	oatA	89.34
66	Uncharacterized protein	Q2FUY8	SAOUHSC_02957	87.34
67	Na(+)/H(+) antiporter subunit G1	Q2G2H9	mnhG1	79.83
68	Uncharacterized protein	Q2G2Y4	SAOUHSC_01918	78.83
69	DNA topoisomerase 4 subunit A	Q2FYS4	parC	75.31
70	Lysostaphin resistance protein A	Q2FVT1	lyrA	75.19
71	Protein translocase subunit SecG	Q2G026	SAOUHSC_00801	74.89
72	Uncharacterized protein	Q2FV90	SAOUHSC_02844	73.66
73	Uncharacterized protein	Q2G0P9	SAOUHSC_00501	71.72
74	Uncharacterized protein	Q2FV15	SAOUHSC_02928	70.34
75	Uncharacterized protein	Q2G2S8	SAOUHSC_01974	68.50
76	Uncharacterized protein	Q2FXF0	SAOUHSC_01899	67.38
77	Protein translocase subunit SecA 1	O06446	secA1	65.48
78	Uncharacterized protein	Q2G0U8	SAOUHSC_00428	65.35
79	Uncharacterized protein	Q2FZ85	SAOUHSC_01155	63.36
80	Signal transduction protein TRAP	Q2G2F3	traP	62.85
81	Fructose specific permease, putative	Q2G239	SAOUHSC_00708	62.40
82	Uncharacterized protein	Q2G2E2	SAOUHSC_00651	60.88
83	Uncharacterized protein	Q2FVN4	SAOUHSC_02668	59.39
84	Uncharacterized protein	Q2FXI4	SAOUHSC_01863	57.78
85	Uncharacterized protein	Q2G1U8	SAOUHSC_02690	55.48
86	Lipase 1	Q2FUU5	lipA	55.27
87	Cell shape-determining protein MreC	Q2FXS6	SAOUHSC_01759	54.69
88	Uncharacterized protein	Q2G161	SAOUHSC_00294	54.15
89	Prolipoprotein diacylglyceryl transferase	Q2G044	lgt	53.90
90	Uncharacterized protein	Q2G242	SAOUHSC_00508	53.35
91	Zinc metalloprotease	Q2G1Z5	SAOUHSC_01239	52.48
92	Uncharacterized protein	Q2FZR7	SAOUHSC_00923	52.27

93	ATP synthase subunit delta	Q2FWE7	atpH	50.37
94	1-acyl-sn-glycerol-3-phosphate acyltransferases domain protein	Q2FXJ7	SAOUHSC_01837	50.00
95	Uncharacterized protein	Q2FVI5	SAOUHSC_02725	48.65
96	PTS system glucoside-specific EIICBA component	Q2FV87	glcB	47.97
97	Uncharacterized protein	Q2G2G6	SAOUHSC_01050	47.61
98	Uncharacterized protein	Q2FZ64	SAOUHSC_01187	46.78
99	Uncharacterized protein	Q2FYL3	SAOUHSC_01427	46.73
100	Oligopeptide ABC transporter, substrate-binding protein, putative	Q2FZR3	SAOUHSC_00927	46.07
101	Amino acid ABC transporter-like protein, putative	Q2FVI1	SAOUHSC_02729	45.44
102	Sodium, sulfate symporter, putative	Q2FUS2	SAOUHSC_03030	45.41
103	Multiple sugar-binding transport ATP-binding protein, putative	Q2G1F0	SAOUHSC_00175	44.62
104	Uncharacterized protein	Q2FVK0	SAOUHSC_02711	42.49
105	Glycerophosphoryl diester phosphodiesterase, putative	Q2FZF9	SAOUHSC_01071	41.88
106	Uncharacterized protein	Q2FXN2	SAOUHSC_01803	41.86
107	Uncharacterized protein	Q2FXF4	SAOUHSC_01895	41.78
108	UDP-N-acetylglucosamine--N-acetylmuramyl-(pentapeptide) pyrophosphoryl-undecaprenol N-acetylglucosamine transferase	Q2FYL5	murG	40.91
109	Methionine import ATP-binding protein MetN 2	Q2FZZ2	metN2	40.48
110	Uncharacterized protein	Q2FX11	SAOUHSC_02096	39.67
111	Uncharacterized protein	Q2FVS2	SAOUHSC_02620	39.58
112	Uncharacterized protein	Q2FYV7	SAOUHSC_01317	38.14
113	ATP synthase epsilon chain	Q2FWF1	atpC	37.76
114	Uncharacterized protein	Q2FZ12	SAOUHSC_01259	37.27
115	Uncharacterized protein	Q2G0B5	SAOUHSC_00690	36.35
116	Uncharacterized protein	Q2FV65	SAOUHSC_02872	33.84
117	Putative antiporter subunit mnhD2	Q2G212	mnhD2	32.96
118	Uncharacterized protein	Q2G2V7	SAOUHSC_02008	32.89
119	Uncharacterized protein	Q2G0R4	SAOUHSC_00482	32.05
120	Ribonuclease Y	Q2FZ08	rny	31.96
121	Phosphatidate cytidylyltransferase	Q2G1Z6	SAOUHSC_01238	31.44
122	Cytochrome d ubiquinol oxidase, subunit I, putative	Q2FZH3	SAOUHSC_01031	31.14
123	Uncharacterized protein	Q2FVF2	SAOUHSC_02762	30.32
124	Quinolone resistance protein NorB	Q2FYJ5	norB	29.56
125	Histidine protein kinase SaeS	Q2G2U1	saeS	28.54
126	Acetyl-CoA acetyltransferase, putative	Q2G1D0	SAOUHSC_00195	27.65

127	Uncharacterized protein	Q2G245	SAOUHSC_01854	27.28
128	Spermidine/putrescine import ATP-binding protein PotA	Q2G2A7	potA	26.50
129	Uncharacterized protein	Q2G0Z4	SAOUHSC_00367	25.55
130	Uncharacterized protein	Q2FVZ5	SAOUHSC_02525	25.16
131	Uncharacterized protein	Q2G074	SAOUHSC_00746	24.96
132	Glycerol-3-phosphate transporter	Q2G138	SAOUHSC_00317	24.71
133	Signal peptidase I	Q2FZT8	SAOUHSC_00902	24.21
134	Rhomboid family protein	Q2FY24	SAOUHSC_01649	22.14
135	Uncharacterized protein	Q2G185	essB	21.92
136	Uncharacterized protein	Q2FW92	dacA	21.15
137	Dihydroorotate dehydrogenase (quinone)	Q2FV30	SAOUHSC_02909	20.91
138	PTS system, glucose-specific IIBC component, putative	Q2G219	SAOUHSC_00209	20.85
139	Uncharacterized protein	Q2G134	SAOUHSC_00322	20.39
140	Uncharacterized protein	Q2FZ00	SAOUHSC_01271	20.18
141	Regulatory protein MsrR	Q7BHL7	msrR	19.55
142	Uncharacterized protein	Q2FZQ4	SAOUHSC_00946	19.49
143	Na(+)/H(+) antiporter subunit D1	Q2G2H7	mnhD1	19.44
144	Uncharacterized protein	Q2FWX2	SAOUHSC_02149	19.21
145	Uncharacterized protein	Q2FZR6	SAOUHSC_00924	18.41
146	Uncharacterized protein	Q2G1M2	SAOUHSC_00085	18.38
147	Transporter, putative	Q2FVE1	SAOUHSC_02773	17.76
148	GTPase Era	Q2FY06	era	17.39
149	Penicillin-binding protein 1	Q2FZ94	SAOUHSC_01145	17.10
150	Uncharacterized protein	Q2FVV1	SAOUHSC_02590	17.07
151	CDP-diacylglycerol--glycerol-3-phosphate 3-phosphatidyltransferase	Q2FZ11	SAOUHSC_01260	15.72
152	Drug resistance transporter, EmrB/QacA subfamily, putative	Q2G2W1	SAOUHSC_02629	15.47
153	Uncharacterized protein	Q2G2T6	SAOUHSC_00015	15.41
154	Cytochrome d ubiquinol oxidase, subunit II, putative	Q2FZH2	SAOUHSC_01032	15.37
155	Uncharacterized protein	Q2FVS4	SAOUHSC_02618	15.07
156	Uncharacterized protein	Q2FXZ8	SAOUHSC_01677	14.99
157	Sodium/glutamate symporter	Q2FVS0	SAOUHSC_02622	14.42
158	Membrane spanning protein, putative	Q2FVB3	SAOUHSC_02821	14.21
159	Teichoic acids export ATP-binding protein TagH	Q2G2L1	tagH	12.91
160	Hydrolase, alpha/beta fold family domain protein	Q2FYZ3	SAOUHSC_01279	12.61
161	Spermidine/putrescine ABC transporter, spermidine/putrescine-binding protein, putative	Q2G2A8	SAOUHSC_01049	12.45

162	Uncharacterized protein	Q2FW93	SAOUHSC_02406	12.31
163	Serine protease HtrA-like	Q2FZP2	SAOUHSC_00958	11.87
164	Copper-exporting P-type ATPase A	Q2FV64	copA	10.85
165	Teichoic acid biosynthesis protein F, putative	Q2G1C1	tarF	10.51
166	Phosphate transport system permease protein PstA	Q2FYP9	SAOUHSC_01386	10.41
167	Uncharacterized protein	Q2FWH2	SAOUHSC_02319	10.12
168	Uncharacterized protein	Q2G2E5	SAOUHSC_00647	9.53
169	Uncharacterized protein	Q2G2Y3	SAOUHSC_01919	9.35
170	Uncharacterized protein	Q2FUS5	SAOUHSC_03026	9.15
171	Sensor protein SrrB	Q2FY80	srrB	8.20
172	Undecaprenyl-diphosphatase	Q2G0B4	uppP	8.10
173	Penicillin-binding protein 3	Q2FY21	SAOUHSC_01652	7.02
174	PTS system, mannitol-specific component, putative	Q2FW99	SAOUHSC_02400	6.83
175	Uncharacterized protein	Q2G1R0	SAOUHSC_00042	6.74
176	Uncharacterized protein	Q2G1P4	SAOUHSC_00060	5.58
177	Uncharacterized protein	Q2FX10	SAOUHSC_02097	5.51
178	Lipoteichoic acid synthase	Q2G093	ltaS	4.75
179	Uncharacterized protein	Q2FY00	SAOUHSC_01675	4.41
180	Phosphonate ABC transporter, substrate-binding protein, putative	Q2G1L7	SAOUHSC_00105	4.23
181	Uncharacterized protein	Q2FXS4	SAOUHSC_01761	3.80
182	Uncharacterized protein	Q2G2H3	SAOUHSC_02009	3.22
183	Uncharacterized protein	Q2FZ18	SAOUHSC_01253	3.06
184	Membrane protein, putative	Q2G1C5	SAOUHSC_00200	2.93
185	Amino acid ABC transporter, permease protein, putative	Q2G088	SAOUHSC_00732	2.91
186	Polysaccharide biosynthesis protein, putative	Q2FXH6	SAOUHSC_01871	2.53
187	Cardiolipin synthase	Q2FWG8	SAOUHSC_02323	2.48
188	Cell division protein DivIB	Q2FZ91	divIB	2.38
189	Pts system, lactose-specific IIBC component, putative	Q2G2D4	SAOUHSC_02450	2.31
190	Extracellular matrix-binding protein ebh	Q2FYJ6	ebh	1.87
191	Elongation factor 4	Q2FXY7	lepA	1.83
192	Uncharacterized protein	Q2FWI3	SAOUHSC_02308	1.50
193	Alanine dehydrogenase 2	Q2FXL7	ald2	1.42
194	Uncharacterized protein	Q2G188	esaA	0.64
195	Protein translocase subunit SecA 2	Q2FUW6	secA2	0.64

Cytoplasm

1	Pyruvate dehydrogenase complex, E1 component, pyruvate dehydrogenase beta subunit, putative	Q2G2A5	SAOUHSC_01041	7139.57
2	Dihydrolipoamide S-acetyltransferase component of pyruvate dehydrogenase complex E2, putative	Q2G2A4	SAOUHSC_01042	6982.71
3	Pyruvate dehydrogenase complex, E1 component, alpha subunit, putative	Q2FZG4	SAOUHSC_01040	6003.62
4	Dihydrolipoyl dehydrogenase	Q2G2A3	SAOUHSC_01043	5493.42
5	Elongation factor Tu	Q2G0N0	tuf	2048.06
6	Dihydrolipoyllysine-residue succinyltransferase component of 2-oxoglutarate dehydrogenase complex	Q2FYM2	odhB	1881.89
7	50S ribosomal protein L1	Q2G0P0	rplA	1860.73
8	50S ribosomal protein L21	Q2FXS8	rplU	1380.56
9	50S ribosomal protein L16	Q2FW13	rplP	1222.07
10	30S ribosomal protein S11	Q2FW31	rpsK	1046.93
11	30S ribosomal protein S4	Q2FXK6	rpsD	834.14
12	50S ribosomal protein L22	Q2FW11	rplV	774.25
13	50S ribosomal protein L2	P60430	rplB	764.74
14	50S ribosomal protein L19	Q2FZ42	rplS	739.30
15	30S ribosomal protein S3	Q2FW12	rpsC	710.15
16	DNA-directed RNA polymerase subunit beta'	Q2G0N5	rpoC	621.39
17	Uncharacterized protein	Q2FVB4	SAOUHSC_02820	579.96
18	Probable malate:quinone oxidoreductase	Q2FV16	mqo	567.87
19	30S ribosomal protein S5	Q2FW23	rpsE	564.05
20	Glutamate dehydrogenase	Q2FZU5	SAOUHSC_00895	546.35
21	2-oxoglutarate dehydrogenase E1 component	Q2FYM1	odhA	522.46
22	50S ribosomal protein L4	Q2FW07	rplD	503.19
23	50S ribosomal protein L20	Q2FXQ1	rplT	479.47
24	50S ribosomal protein L15	P0A0F8	rplO	476.37
25	Aerobic glycerol-3-phosphate dehydrogenase	Q2FYZ4	glpD	471.35
26	DNA-directed RNA polymerase subunit beta	P47768	rpoB	468.87
27	30S ribosomal protein S2	Q2FZ25	rpsB	460.35
28	Cell division protein FtsZ	Q2FZ89	ftsZ	412.33
29	Uncharacterized protein	Q2G2L2	SAOUHSC_00637	366.98
30	30S ribosomal protein S9	Q2FW39	rpsI	361.78
31	Putative universal stress protein SAOUHSC_01819	Q2FXL6	SAOUHSC_01819	343.50
32	Probable glycine dehydrogenase (decarboxylating) subunit 1	Q2FY34	gcvPA	330.28
33	NADH dehydrogenase-like protein SAOUHSC_00878	Q2FZV7	SAOUHSC_00878	323.25
34	30S ribosomal protein S12	P0A0H0	rpsL	283.61

35	Elongation factor G	Q2G0N1	fusA	274.61
36	Enolase	Q2G028	eno	272.29
37	DEAD-box ATP-dependent RNA helicase CshA	Q2FWH5	cshA	247.32
38	50S ribosomal protein L5	Q2FW18	rplE	247.31
39	30S ribosomal protein S7	P48940	rpsG	241.90
40	Ribonuclease J 1	Q2FZG9	rnj1	241.40
41	Glyceraldehyde-3-phosphate dehydrogenase	Q2G032	SAOUHSC_00795	237.61
42	50S ribosomal protein L10	Q2G0N9	rplJ	236.55
43	Glutamine synthetase	Q2FYY6	SAOUHSC_01287	235.00
44	Pyruvate kinase	Q2FXM9	pyk	228.14
45	Alkyl hydroperoxide reductase subunit C	P0A0B7	ahpC	202.08
46	Probable glycine dehydrogenase (decarboxylating) subunit 2	Q2FY35	gcvPB	201.69
47	UPF0637 protein SAOUHSC_01054	Q2G2G7	SAOUHSC_01054	193.94
48	3-hydroxyacyl-[acyl-carrier-protein] dehydratase FabZ	Q2FWF5	fabZ	191.00
49	Bacterial non-heme ferritin	Q2FWZ8	ftnA	187.16
50	DNA-binding protein HU, putative	Q2FYG2	SAOUHSC_01490	179.25
51	Uncharacterized protein	Q2FXM1	SAOUHSC_01814	178.02
52	50S ribosomal protein L6	Q2FW21	rplF	168.63
53	DEAD-box ATP-dependent RNA helicase CshB	Q2FY15	cshB	164.22
54	1,4-dihydroxy-2-naphthoyl-CoA synthase	Q2FZL5	menB	162.81
55	Putative aldehyde dehydrogenase AldA	Q2G1J0	aldA	161.75
56	Pyridoxal 5'-phosphate synthase subunit PdxS	Q2G0Q1	pdxS	160.29
57	Asparagine--tRNA ligase	Q2FYH6	asnS	157.55
58	Uncharacterized protein	Q2G1B7	SAOUHSC_00228	152.25
59	Uncharacterized protein	Q2FWB7	SAOUHSC_02381	145.81
60	Inosine-5'-monophosphate dehydrogenase	Q2G0Y7	guaB	145.05
61	DNA-directed RNA polymerase subunit alpha	Q2FW32	rpoA	142.49
62	Glycine--tRNA ligase	Q2FY08	glyQS	140.42
63	Glutamyl-tRNA(Gln) amidotransferase subunit A	Q2FWY9	gatA	138.84
64	Ribose-phosphate pyrophosphokinase	Q2G0S2	prs	134.13
65	Ribonuclease J 2	Q2FZ19	rnj2	130.76
66	50S ribosomal protein L3	Q2FW06	rplC	127.02
67	Signal recognition particle protein	Q2FZ46	ffh	126.41
68	Enoyl-[acyl-carrier-protein] reductase [NADPH] FabI	Q2FZQ3	fabI	126.11
69	Menaquinone biosynthesis methyltransferase, putative	Q2FYG5	SAOUHSC_01487	125.17
70	Uncharacterized protein	Q2G1C8	SAOUHSC_00197	115.64

71	Translation initiation factor IF-2	Q2G2D0	infB	115.45
72	Probable malate:quinone oxidoreductase	Q2FVQ5	mqo	115.17
73	30S ribosomal protein S13	Q2FW30	rpsM	114.58
74	Uncharacterized protein	Q2FVW3	SAOUHSC_02579	111.22
75	Formate--tetrahydrofolate ligase	Q2G296	fhs	110.02
76	50S ribosomal protein L14	Q2FW16	rplN	109.77
77	HTH-type transcriptional regulator SarS	Q2G1N7	sarS	105.20
78	ABC transporter, ATP-binding protein, putative	Q2FWW9	SAOUHSC_02152	99.55
79	Isocitrate dehydrogenase [NADP]	Q2FXN4	SAOUHSC_01801	96.76
80	Glycerol kinase	Q2FYZ5	glpK	93.55
81	Translation initiation factor IF-3	Q2FXP9	infC	92.96
82	Uncharacterized protein	Q2G2L3	tarA	92.05
83	Uncharacterized protein	Q2FVL6	SAOUHSC_02695	90.62
84	Uncharacterized protein	Q2FZ59	SAOUHSC_01192	88.18
85	Amino acid ABC transporter, ATP-binding protein, putative	Q2FVL4	SAOUHSC_02697	87.57
86	Proline--tRNA ligase	Q2G1Z4	proS	86.79
87	Chaperone protein DnaK	Q2FXZ2	dnaK	81.67
88	1-pyrroline-5-carboxylate dehydrogenase	Q2FV67	rocA	78.08
89	30S ribosomal protein S8	Q2FW20	rpsH	74.98
90	50S ribosomal protein L25	Q2G0S0	rplY	74.82
91	Acetate kinase	Q2FXL5	ackA	73.76
92	Uncharacterized protein	Q2FY81	SAOUHSC_01584	72.60
93	Lipid II:glycine glycyltransferase	Q2FVZ4	femX	72.60
94	GTP-sensing transcriptional pleiotropic repressor CodY	Q2FZ27	codY	71.52
95	Uncharacterized protein	Q2G2S7	SAOUHSC_01975	70.98
96	50S ribosomal protein L13	Q2FW38	rplM	69.80
97	Serine hydroxymethyltransferase	Q2FWE5	glyA	69.79
98	Pur operon repressor	Q2G0S7	SAOUHSC_00467	69.57
99	Probable DNA-directed RNA polymerase subunit delta	Q2FWD0	rpoE	68.95
100	Uncharacterized protein	Q2FZR5	SAOUHSC_00925	68.31
101	Aminoacyltransferase FemA	Q2FYR2	femA	67.53
102	Protein translocase subunit SecA 1	O06446	secA1	65.48
103	Oligopeptide ABC transporter, ATP-binding protein, putative	Q2FZR4	SAOUHSC_00926	64.78
104	Succinyl-CoA ligase [ADP-forming] subunit alpha	Q2FZ36	sucD	64.23
105	Uncharacterized protein	Q2FV35	SAOUHSC_02904	62.61
106	Succinyl-CoA ligase [ADP-forming] subunit beta	Q2FZ37	sucC	61.95

107	Iron compound ABC transporter, substrate-binding protein, putative	Q2G0F6	SAOUHSC_00613	61.17
108	Ornithine aminotransferase	Q2FZU6	rocD	59.15
109	CTP synthase	Q2FWD1	pyrG	57.72
110	30S ribosomal protein S19	Q2FW10	rpsS	57.66
111	Uncharacterized protein	Q2FYN0	SAOUHSC_01405	56.27
112	Glycosyl transferase, group 1	Q2FZM6	SAOUHSC_00974	55.98
113	Aminomethyltransferase	Q2FY33	gcvT	52.75
114	30S ribosomal protein S15	Q2G2Q1	rpsO	52.45
115	ABC transporter, ATP-binding protein, putative	Q2G1V4	SAOUHSC_00333	50.48
116	Adapter protein MecA	Q2G1U5	mecA	49.55
117	30S ribosomal protein S18	Q2G111	rpsR	48.17
118	Uncharacterized protein	Q2FVW0	SAOUHSC_02581	46.39
119	Uncharacterized protein	Q2G2W3	SAOUHSC_02627	44.78
120	Uncharacterized protein	Q2FX98	SAOUHSC_01979	43.21
121	Uncharacterized protein	Q2FWD3	SAOUHSC_02366	40.96
122	ABC transporter domain protein	Q2G089	SAOUHSC_00731	40.38
123	Uncharacterized protein	Q2FVS3	SAOUHSC_02619	38.84
124	Uncharacterized protein	Q2FXX9	SAOUHSC_01701	37.99
125	50S ribosomal protein L18	Q2FW22	rplR	37.41
126	Ribosome hibernation promotion factor	Q2G055	hpf	36.97
127	Holliday junction ATP-dependent DNA helicase RuvA	Q2FXT3	ruvA	35.78
128	50S ribosomal protein L7/L12	P48860	rplL	35.12
129	Acetyl-CoA carboxylase, biotin carboxylase, putative	Q2FXX1	SAOUHSC_01709	34.74
130	Glutamate synthase alpha subunit, putative	Q2FVF4	SAOUHSC_02760	34.56
131	50S ribosomal protein L23	Q2FW08	rplW	34.47
132	Aspartyl/glutamyl-tRNA(Asn/Gln) amidotransferase subunit B	Q2FWZ0	gatB	34.35
133	Transcription termination factor Rho	Q2FWD7	rho	34.04
134	Uncharacterized protein	Q2FXW9	SAOUHSC_01711	33.91
135	Peptide methionine sulfoxide reductase MsrB	P0A088	msrB	33.60
136	Glutamine--fructose-6-phosphate aminotransferase [isomerizing]	Q2FWA0	glmS	33.43
137	Catalase	Q2FYU7	katA	32.68
138	HTH-type transcriptional regulator SarR	Q9F0R1	sarR	32.57
139	Formate acetyltransferase	Q2G1D8	pflB	32.50
140	Putative formate dehydrogenase SAOUHSC_02582	Q2FVV9	SAOUHSC_02582	32.36
141	Pyrimidine-nucleoside phosphorylase	Q2FWC1	pdp	31.97
142	Fructose-bisphosphate aldolase class 1	Q2FV17	fda	30.20

143	Uncharacterized protein	Q2G0F3	SAOUHSC_00616	30.06
144	Ferredoxin, putative	Q2FYE8	SAOUHSC_01504	28.94
145	Uncharacterized protein	Q2G0D6	SAOUHSC_00669	28.17
146	Uncharacterized protein	Q2FY44	SAOUHSC_01622	28.08
147	Uracil phosphoribosyltransferase	Q2FWE6	upp	27.98
148	Uncharacterized protein	Q2FZM7	SAOUHSC_00973	27.93
149	Conserved virulence factor B	Q2FYP3	cvfB	27.02
150	3-oxoacyl-(Acyl-carrier-protein) reductase, putative	Q2FZ53	SAOUHSC_01199	25.71
151	Pyruvate oxidase, putative	Q2FV86	SAOUHSC_02849	25.57
152	Lysine--tRNA ligase	Q2G0Q3	lysS	25.33
153	HTH-type transcriptional regulator SarX	Q2G0D1	sarX	25.11
154	Uncharacterized protein	Q2FZW0	SAOUHSC_00875	24.71
155	Ribonucleoside-diphosphate reductase	Q2G078	SAOUHSC_00742	24.47
156	Phosphate acyltransferase	Q2FZ55	plsX	23.78
157	Nucleoside diphosphate kinase	Q2FYG7	ndk	22.96
158	RNA polymerase sigma factor SigA	P0A0J0	sigA	22.93
159	30S ribosomal protein S1, putative	Q2FYF9	SAOUHSC_01493	22.12
160	2-oxoisovalerate dehydrogenase, E2 component, dihydrolipoamide acetyltransferase, putative	Q2FY54	SAOUHSC_01611	21.31
161	Dihydroxy-acid dehydratase	Q2FWK7	ilvD	20.65
162	Protein RecA	Q2FZ09	recA	20.15
163	Uncharacterized protein	Q2FYK7	SAOUHSC_01433	19.57
164	Ribosomal RNA small subunit methyltransferase H	P60393	rsmH	18.46
165	HMG-CoA synthase, putative	Q2FV76	SAOUHSC_02860	18.38
166	Aminoacyltransferase FemB	Q2FYR1	femB	18.30
167	Uncharacterized protein	Q2FZH1	SAOUHSC_01034	18.21
168	ATP-dependent Clp protease ATP-binding subunit ClpX	Q2FXQ7	clpX	17.98
169	Aconitate hydratase	Q2FYS9	SAOUHSC_01347	17.90
170	Bifunctional protein FolD	Q2FZJ6	folD	17.90
171	GTPase Era	Q2FY06	era	17.39
172	Elongation factor Ts	Q2FZ23	tsf	16.64
173	GTPase Obg	Q2FXT1	obg	16.63
174	Uncharacterized protein	Q2FXH5	SAOUHSC_01872	16.59
175	Superoxide dismutase [Mn] 1	P0A0J3	sodA	16.45
176	UDP-N-acetylglucosamine 1-carboxyvinyltransferase	Q2FWF4	murA	16.04
177	Iron-sulfur cluster carrier protein	Q2FW86	SAOUHSC_02417	15.34
178	Trigger factor	Q2FXQ6	tig	14.55

179	Uncharacterized protein	Q2FVV6	SAOUHSC_02585	13.64
180	Uncharacterized protein	Q2G1C9	SAOUHSC_00196	13.43
181	Aldehyde dehydrogenase	Q2FWX9	SAOUHSC_02142	13.42
182	Ribosomal RNA small subunit methyltransferase B	Q2FZ67	SAOUHSC_01184	12.82
183	Glucose-6-phosphate isomerase	Q2FZU0	pgi	12.67
184	Acetyl-coenzyme A carboxylase carboxyl transferase subunit beta	Q2FXM6	accD	12.44
185	Uridylate kinase	Q2FZ22	pyrH	12.41
186	Cysteine synthase	Q2G0Q8	SAOUHSC_00488	12.10
187	Glyceraldehyde-3-phosphate dehydrogenase	Q2FXP2	SAOUHSC_01794	10.54
188	Uncharacterized protein	Q2G0Z2	SAOUHSC_00369	10.35
189	Hypoxanthine phosphoribosyltransferase	Q2G0R1	SAOUHSC_00485	10.04
190	Catabolite control protein A	Q2G295	SAOUHSC_01850	9.47
191	Uncharacterized protein	Q2FXD3	SAOUHSC_01930	9.41
192	Ribonucleotide-disphosphate reductase beta chain, putative	Q2G077	SAOUHSC_00743	9.37
193	Elongation factor P	Q2FY41	efp	9.30
194	60 kDa chaperonin	Q2FWN4	groL	9.21
195	3-hydroxy-3-methylglutaryl coenzyme A reductase	Q2FV77	SAOUHSC_02859	8.78
196	DNA topoisomerase 4 subunit B	Q2FYS5	parE	8.70
197	Uncharacterized protein	Q2FX90	SAOUHSC_01987	8.33
198	Ribonuclease R	Q2G024	rnr	8.27
199	S1 RNA binding domain protein	Q2FWJ5	SAOUHSC_02297	8.26
200	DNA topoisomerase 1	Q2FZ32	topA	7.89
201	ABC transporter, ATP-binding protein, putative	Q2G2F1	SAOUHSC_01967	7.32
202	Uncharacterized protein	Q2G0M8	SAOUHSC_00532	7.30
203	TelA-like protein SAOUHSC_01408	Q2FYM7	SAOUHSC_01408	7.23
204	HTH-type transcriptional regulator rot	Q9RFJ6	rot	7.21
205	Alanine--tRNA ligase	Q2FXV9	alaS	7.08
206	3-oxoacyl-[acyl-carrier-protein] synthase 2	Q2FZR9	SAOUHSC_00921	6.76
207	L-lactate dehydrogenase 2	Q2G1Y5	ldh2	6.74
208	Leucine aminopeptidase 2, chloroplastic	Q2FZV6	SAOUHSC_00879	6.11
209	Type I restriction-modification system, M subunit	Q2FXD0	SAOUHSC_01933	6.02
210	Uncharacterized protein	Q2FXY2	SAOUHSC_01698	5.99
211	Uncharacterized protein	Q2G0L2	SAOUHSC_00548	5.48
212	Uncharacterized protein	Q2FXA7	SAOUHSC_01958	5.40
213	ATP-dependent 6-phosphofructokinase	Q2FXM8	pfkA	5.13
214	Uncharacterized protein	Q2G2J9	SAOUHSC_01412	4.86

215	Uncharacterized protein	Q2FWC6	SAOUHSC_02372	4.80
216	Urocanate hydratase	Q2FVT5	hutU	4.58
217	DNA gyrase subunit A	Q2G2Q0	gyrA	4.40
218	Heptaprenyl diphosphate syntase component II, putative	Q2FYG6	SAOUHSC_01486	4.39
219	DNA polymerase III, gamma and tau subunits, putative	Q2G0T5	dnaX	3.78
220	Oxygen-dependent choline dehydrogenase	Q2FV11	betA	3.38
221	Uncharacterized protein	Q2FXI9	SAOUHSC_01857	3.04
222	Ribosome-binding ATPase YchF	Q2G115	ychF	2.91
223	Fructose-1,6-bisphosphatase class 3	Q2FVB2	fbp	2.84
224	Polyribonucleotide nucleotidyltransferase	Q2FZ20	pnp	2.62
225	ATP-dependent protease ATPase subunit HslU	Q2FZ28	hslU	2.37
226	GTP-binding protein TypA, putative	Q2G1Y6	SAOUHSC_01058	2.25
227	Adenylosuccinate lyase	Q2G2S0	purB	2.25
228	Fumarate hydratase class II	Q2FX94	fumC	2.16
229	Uncharacterized protein	Q2FVQ0	SAOUHSC_02652	2.16
230	Uncharacterized protein	Q2G146	SAOUHSC_00309	1.94
231	S-adenosylmethionine synthase	Q2G1W4	metK	1.93
232	D-alanine aminotransferase	Q2FXI0	SAOUHSC_01867	1.74
233	D-lactate dehydrogenase, putative	Q2FV62	SAOUHSC_02875	1.69
234	Phosphopantothenoylcysteine decarboxylase/phosphopantothenate--cysteine ligase	Q2G268	SAOUHSC_01178	1.54
235	Uncharacterized protein	Q2FYJ0	SAOUHSC_01455	1.50
236	DNA polymerase	Q2FXN9	SAOUHSC_01797	1.41
237	Transcription-repair-coupling factor	Q2G0R8	mfd	1.41
238	Uncharacterized protein	Q2FXX4	SAOUHSC_01706	1.41
239	NADP-dependent malic enzyme, putative	Q2FXM5	SAOUHSC_01810	1.31
240	UDP-N-acetylmuramyl tripeptide synthetase, putative	Q2FWZ9	SAOUHSC_02107	1.23
241	Threonine--tRNA ligase	Q2FXP7	thrS	0.89
242	DNA ligase	Q2G1Y0	ligA	0.72
243	Protein translocase subunit SecA 2	Q2FUW6	secA2	0.64

[a] The proteins are ordered from the highest to lowest APEX score. APEX normalization factor = 100,000.

Supplementary Methods

IL-8 assay. HEK293 cells were transfected either with pDUO-mock or pDUO-human TLR4/MD2 (Invivogen) according to the manufacturer's instruction[1]. The transfected cells were seeded on a 24-well plate (1x10^5 cells/well). Various concentrations of *E. coli* wild-type or *ΔmsbB* OMVs (0, 10, 100, or 1000 ng/ml) were treated to the cells with 0.5% FBS, and the culture supernatants were harvested at 6 h after treatment. The concentration of IL-8 was measured in the culture supernatants by DuoSet ELISA kit (R&D Systems).

S. aureus **wildtype extracellular vesicle targeting** ***in vivo***. *S. aureus* wildtype extracellular vesicles were labeled with Cy7 mono NHS ester (Amersham Biosciences) by 2 h incubation at 37 °C. Excess Cy7 was removed using ultracentrifugation at 150,000*g* for 3 h at 4°C. Cy7-labeled extracellular vesicles (10 μg in total protein) were injected intravenously to normal mice and mice bearing tumor with a diameter of 15 mm (male, 8 weeks old). Mice were anesthetized and shaved before the intravenous injection of Cy7-labeled OMVs. Cy7 signals were measured using the IVIS spectrum (Caliper Life Sciences), 12 h after the injection.

IFN-γ neutralization. For IFN-γ neutralization, 200 μg of mouse monoclonal anti-IFN-γ IgG1 (Bio X Cell, BE0055) was intraperitoneally injected 24 h before each OMV injection. Control group was intraperitoneally injected with 200 μg of isotype IgG_1 antibody (Bio X Cell, BE0290) 24 h before each OMV injection.

Quantification of IFN-γ after heated and trypsin-treated extracellular vesicles injection. To prepare heated bacterial extracellular vesicles, *E. coli* Δ*msbB* OMVs and *S. aureus* WT extracellular vesicles were boiled at 100°C for 10 min. For trypsin treatment, *E. coli* Δ*msbB* OMVs and *S. aureus* WT extracellular vesicles were treated with trypsin (Promega) at 37°C

for 1 h and was added with trypsin inhibitor cocktail and excess buffer. The trypsin-treated extracellular vesicles were collected by ultracentrifugation at 150,000*g* for 2 h at 4°C. Then, the extracellular vesicle samples were intravenously injected to mice bearing tumors with a diameter of around 15 mm and serum were collected after 6 h and tumor tissues were collected after 48 h for IFN-γ measurement.

Proteomic analysis on extracellular vesicles. To identify the proteomes of extracellular vesicles derived from *E. coli* W3110 *ΔmsbB* and wildtype *S. aureus*, in-solution digestion was performed with filter-aided sample preparation[2]. The vesicular proteins (50 μg) were solubilized in lysis buffer (50 mM Tris-HCl (pH 7.5), 1% NP-40, 0.25% sodium deoxycholate, 100 mM sodium chloride, 1 mM EDTA, and protease inhibitor cocktail (Roche Applied Science) and then denatured with 6 M guanidine-HCl and reduced with 5 mM Tris (2-carboxyethyl) phosphine hydrochloride (Thermo Scientific) for 5 min at 95ºC. The denatured proteins were alkylated with 50 mM iodoacetoamide for 30 min in the dark at room temperature. After alkylation, vesicular proteins were concentrated by methanol/chloroform precipitation[3]. Precipitated proteins were resuspended with 2 M urea in 50 mM NH_4HCO_3 and digested with sequencing-grade modified trypsin (enzyme to protein ratio 1:100; Promega) for 16 h at 37ºC. The tryptic peptides were loaded on Amicon Ultra 10K filters (Millipore) and collected by centrifugation at 14,000*g* for 20 min. The residual concentrates in the filter were additionally digested with trypsin (enzyme to protein ratio 1:100) in 50 mM NH_4HCO_3 for 6 h at 37ºC. Residual tryptic peptides were collected by centrifugation at 14,000*g* for 20 min at 4ºC. Finally, the filters were rinsed with 500 mM sodium chloride and centrifuged at 14,000*g* for 20 min at 4ºC. All eluents from the filters were collected and desalted with C18 column (Thermo Scientific) for mass spectrometry.

The desalted tryptic peptides were analyzed with an LTQ-Orbitrap Velos mass spectrometer (Thermo Finnigan) combined with EASY-nLC II (Thermo Finnigan). The tryptic peptides were separated using a lab-made microcapillary column (75 μm × 12 cm) packed with C18 resin with an average diameter of 5 μm (Michrom Bioresources). The sample was separated using a linear 60 min gradient of a mixture of solvent A (0.1% formic acid in 2% acetonitrile) to 60% solvent B (0.1% formic acid in 98% acentonitrile) at a flow rate of 300 nL/min. The separated peptides were electrosparyed with the electrospray voltage of 2.6 kV. All MS/MS spectra were obtained in data-dependent scans for fragmentation of the ten most abundant spectra from full scans. We used the following dynamic exclusion conditions to increase the size of proteome to be detected: dynamic exclusion duration = 180 sec, exclusion mass width = 1.5 Da, repeat count for dynamic exclusion = 1, repeat duration = 30 sec, and the list size of dynamic exclusion = 50.

Five and nine technical replicates of LC-MS/MS data of *E. coli* Δ*msbB* OMVs and *S. aureus* extracellular vesicles were analysed using X!Tandem with Trans-Proteomic Pipeline (version 4.8.0) [4], respectively. Using MSConverter, RAW files were converted into mzXML files. The mzXML files from mass spectrometry of extracellular vesicles derived from *E. coli* and *S. aureus* were searched in the reference proteome databases of *E. coli* (strain K-12; 4,307 entries) and *S. aureus* database (strain NCTC8325; 2,889 entries) from UniProt database (release 2017_04), respectively, using X!Tandem. To check contamination from the culture media, which contain yeast extracts and tryptone (peptides formed by the digestion of casein by trypsin), the mzXML files were also search in the reference proteomes of yeast (*Saccharomyces cerevisiae* strain ATCC 204508; 6,721 entries) and cow (*Bos taurus*; 24,148 entries). Tolerance was set to 10 ppm and 0.8 Da for precursor ions and fragment ions,

respectively. Enzyme specificity was set to trypsin, and the number of missed cleavage sites was set to two. Four variable modification options were used: carbamidomethylation of cysteine (+57.021 Da), deamination of N-terminal glutamine (-17.027 Da), dehydration of N-terminal glutamic acid (-18.011 Da), and oxidation of methionine (+15.995 Da). Trans-Proteomic Pipeline was employed to statistically identify peptides by PeptideProphet (PeptideProphet $\geq$ 0.9) and proteins by ProteinProphet (ProteinProphet $\geq$ 0.9). The relative abundance of each vesicular protein was quantitated by absolute protein expression (APEX; version 1.1.0)[5]. To generate an experiment-specific dataset, a training dataset was selected from the first 50 top-ranked proteins with high spectral counts from ProteinProphet results. APEX normalization factor was set to 100,000, and the relative abundance of each vesicular protein was quantitated as previously reported[5].

Subcellular localizations of vesicular proteins were acquired from UniProt (release 2017_04)[6,7], DAVID[8,9], and LocateP[10] databases. The statistical significance between cellular and extracellular vesicle proteomes was calculated using Fisher's exact test[11].

Supplementary References

1 Pugin, J. *et al*. Soluble MD-2 activity in plasma from patients with severe sepsis and septic shock. *Blood* **104**, 4071-4079, doi: 10.1182/blood-2003-04-1290 (2004).

2 Wisniewski JR, Zougman A, Nagaraj N, Mann M. Universal sample preparation method for proteome analysis. *Nat Methods* **6**, 359-362 (2009).

3 Wessel D, Flugge UI. A method for the quantitative recovery of protein in dilute solution in the presence of detergents and lipids. *Anal Biochem* **138**, 141-143 (1984).

4 Keller A, Eng J, Zhang N, Li XJ, Aebersold R. A uniform proteomics MS/MS analysis platform utilizing open XML file formats. *Mol Syst Biol* **1**, 2005 0017 (2005).

5 Braisted JC, *et al*. The APEX Quantitative Proteomics Tool: generating protein quantitation estimates from LC-MS/MS proteomics results. *BMC Bioinformatics* **9**, 529 (2008).

6 Apweiler R, *et al*. UniProt: the Universal Protein knowledgebase. *Nucleic Acids Res* **32**, D115-119 (2004).

7 UniProt C. Activities at the Universal Protein Resource (UniProt). *Nucleic Acids Res* **42**, D191-198 (2014).

8 Huang da W, Sherman BT, Lempicki RA. Bioinformatics enrichment tools: paths toward the comprehensive functional analysis of large gene lists. *Nucleic Acids Res* **37**, 1-13 (2009).

9 Huang da W, Sherman BT, Lempicki RA. Systematic and integrative analysis of large gene lists using DAVID bioinformatics resources. *Nat Protoc* **4**, 44-57 (2009).

10 Zhou M, Boekhorst J, Francke C, Siezen RJ. LocateP: genome-scale subcellular-location predictor for bacterial proteins. *BMC Bioinformatics* **9**, 173 (2008).

11 Fisher RA. Statistical methods for research workers (1954).
